# The subclonal structure and genomic evolution of oral squamous cell carcinoma revealed by ultra-deep sequencing

**DOI:** 10.18632/oncotarget.15014

**Published:** 2017-02-02

**Authors:** Siavosh Tabatabaeifar, Mads Thomassen, Martin J Larsen, Stine R Larsen, Torben A Kruse, Jens A Sørensen

**Affiliations:** ^1^ Department of Plastic Surgery, Odense University Hospital, Odense, Denmark; ^2^ Department of Clinical Genetics, Odense University Hospital, Odense, Denmark; ^3^ Department of Clinical Pathology, Odense University Hospital, Odense, Denmark; ^4^ Department of University of Southern Denmark, Institute of Clinical Research, Odense, Denmark

**Keywords:** ultra-deep sequencing, oral squamous cell carcinoma, tumor heterogeneity, subclonal structure, genomic evolution

## Abstract

Recent studies suggest that head and neck squamous cell carcinomas are very heterogeneous between patients; however the subclonal structure remains unexplored mainly due to studies using only a single biopsy per patient. To deconvolute the clonal structure and describe the genomic cancer evolution, we applied whole-exome sequencing combined with ultra-deep targeted sequencing on oral squamous cell carcinomas (OSCC). From each patient, a set of biopsies was sampled from distinct geographical sites in primary tumor and lymph node metastasis.

We demonstrate that the included OSCCs show a high degree of inter-patient heterogeneity but a low degree of intra-tumor heterogeneity. However, some OSCC cancers contain complex subclonal architectures comprising distinct subclones only found in geographically distinct regions of the primary tumors. In several cases we find mutations in the primary tumor that are not present in the lymph node metastasis. We conclude that metastatic potential in our population is acquired early in tumor evolution as evident by the ongoing parallel evolution in several primary tumors.

## INTRODUCTION

Head and neck cancer is the world’s 6th most common cancer form with more than a half million new cases a year. More than 90% of tumors are head and neck squamous cell carcinomas (HNSCC). Recent studies suggest that they are very heterogeneous between patients [1–11]. Oral squamous cell carcinoma (OSCC), a subgroup of HNSCC, is primarily attributed to alcohol consumption and tobacco use. The role of human papilloma virus (HPV) in OSCC is questionable. Recent international studies suggest that despite a higher HPV DNA prevalence than previously reported, HPV rarely plays a driving role in oncogenesis, because mRNA or p16 are detected in only 3% to 5% of oral cavity cancers [12, 13].

OSCC is a loco-regional disease that mainly involves the oral cavity and cervical lymph nodes; distant metastasis is relatively rare in HNSCC compared to other cancer types. In a 2009 study, OSCC was shown to spread less frequently to distant sites compared to other HNSCC tumors localized at oropharynx, laryngopharynx and larynx, 6% vs 16% [14].

Intra-tumor heterogeneity and subclonal structure of OSCC (and HNSCC) have remained unexplored mainly due to studies using only a single biopsy per patient, as the use of a single tumor biopsy severely hinders the analysis of spatial intra-tumor heterogeneity. Analysis of intra-tumor heterogeneity in HNSCC has previously been based on calculating a mutant-allele tumor heterogeneity score [15], which could be useful if only one biopsy is available. In order to avoid this limitation and to obtain a higher resolution, we sampled multiple tumor biopsies from each patient. Another limitation of the previous studies is the relatively low coverage obtained from whole-exome sequencing (WES). To deconvolute the clonal structure and describe the genomic cancer evolution, we applied WES combined with ultra-deep targeted sequencing on OSCCs with cervical lymph node involvement.

## RESULTS

To evaluate the subclonal diversity of OSCC we analyzed three tumor biopsies (named front, center and back, respectively – Supplementary Figure 1) and one lymph node metastasis from 5 late stage patients (Supplementary Table 1) using whole-exome sequencing. Average coverage of WES across all samples was 95×. Possible somatic variants were selected and validated by targeted ultra-deep sequencing with an average coverage of 1693× (Supplementary Table 2). Approximately 80% of the possible variants were confirmed. The number of mutations ranges from 27 to 156 in our population. Our data shows a significantly higher nonsynonymous to synonymous ratio that exceeds the 2:1 ratio [16] one would expect if these were random passenger mutations (*p*-value = *0.0002*, Supplementary Table 9). The primary tumors were tested for p16 protein overexpression by immunohistochemistry which is used as a prognostic marker for HPV infection. Patient 5 was the only positive case.

### Intra-tumor heterogeneity

**Figure 1:**
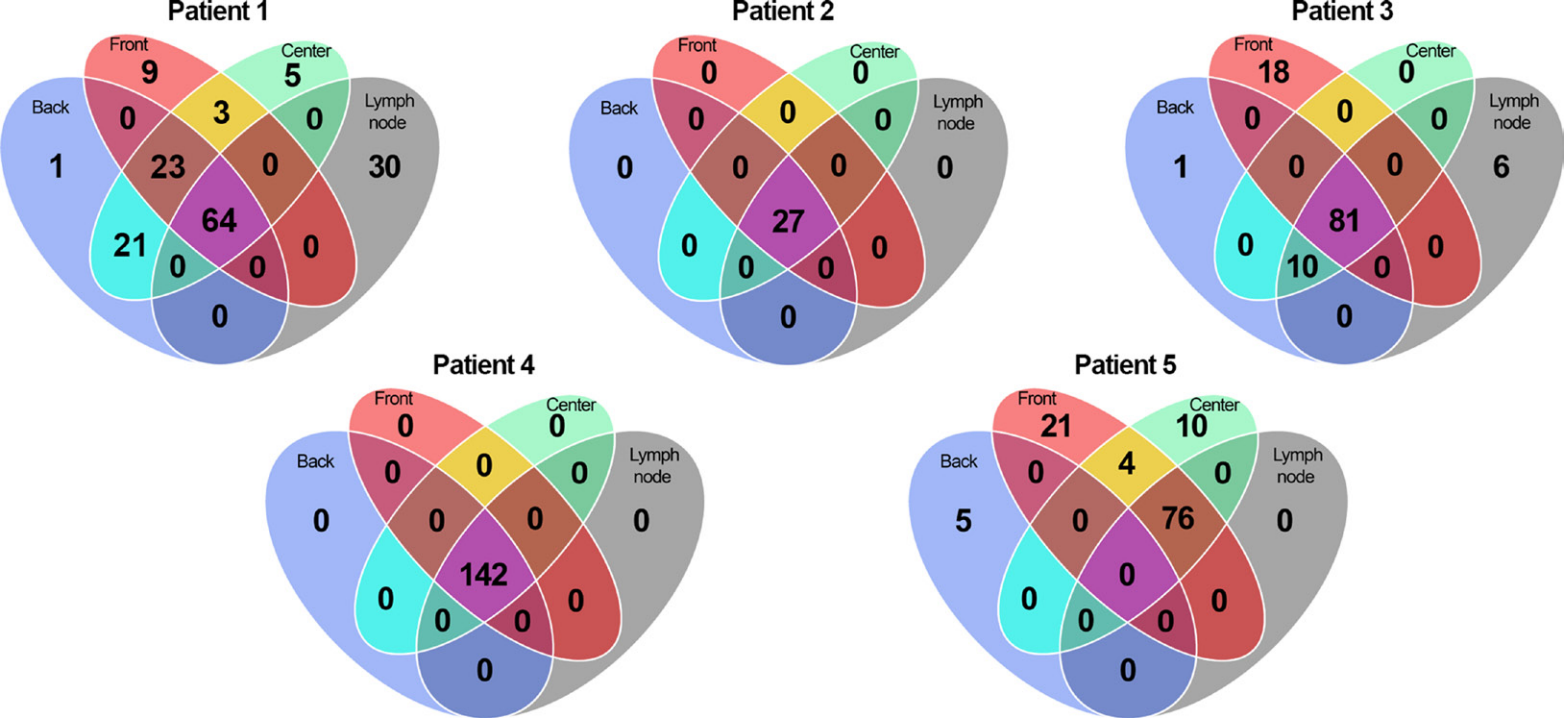
Venn diagrams Venn diagrams for all patients illustrating the shared mutations across biopsies. A mutation has to have sufficiently high enough alternative reads (B-alleles) before being counted (in this case, at least 5). It should be noted that tumor back in patient 5 was evaluated not to contain tumor tissue.

Heterogeneity analysis of the primary tumors demonstrates that biopsies from each patient typically share a common set of somatic mutations which comprises the majority of mutations found (Figure 1). Interestingly, in several cases we find mutations in the primary tumor that are not present in the lymph node metastasis. Lymph node metastasis specific mutations are observed in 2 of the 5 cases.

### Genomic evolution and subclonal structure

**Figure 2:**
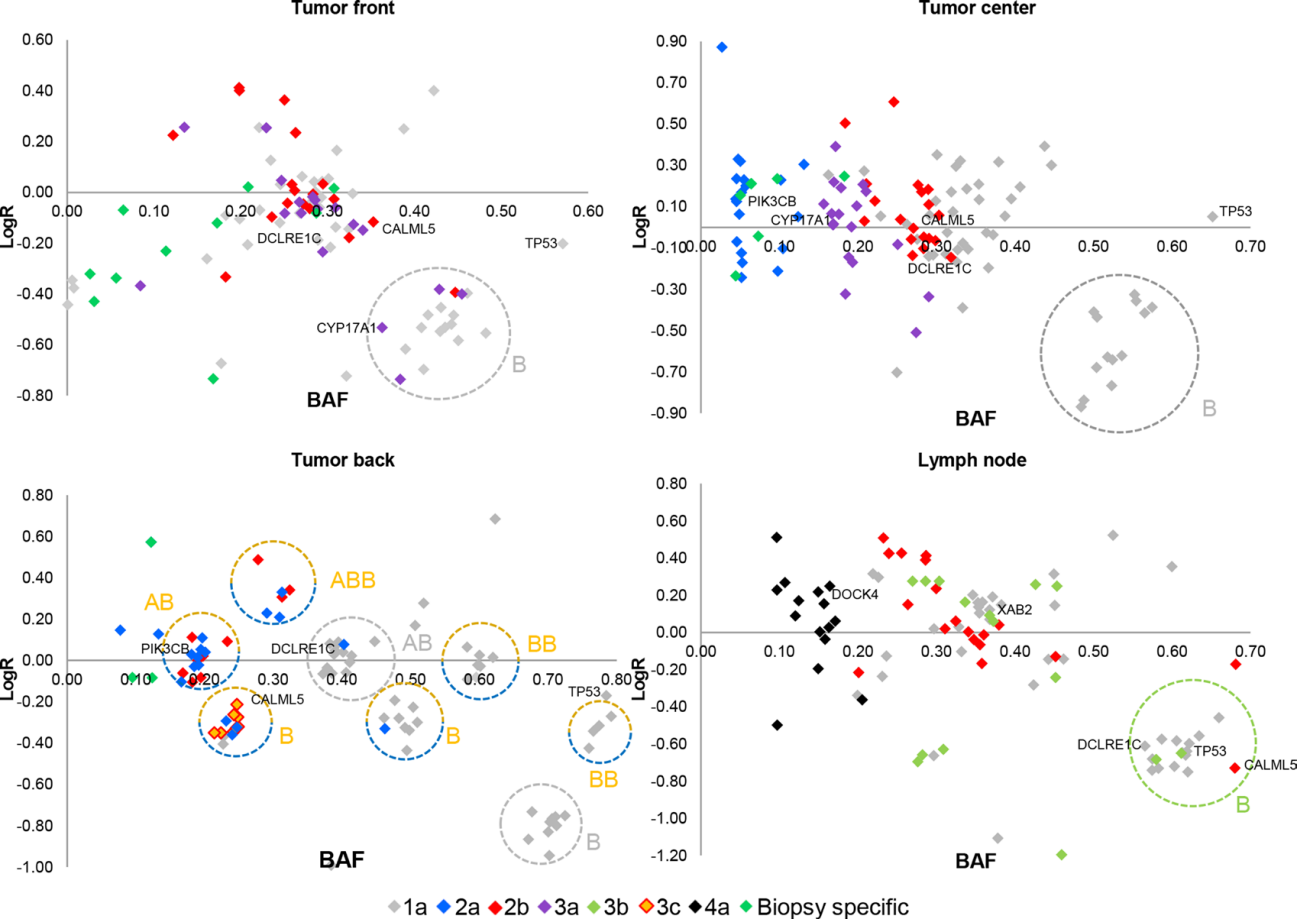
B-Allele frequency (BAF) vs copy number plots for patient 1 LogR is defined as the Log2 copy number ratio between the tumor and matched normal sample. Each point represents a somatic point mutation and each circle represents a copy number event; each point and circle is color-coded according to the clonal structure of the phylogenetic tree (Figure 3). Overall, the bulk of mutations are clustered around a LogR of zero at half of the maximum BAF indicating the heterozygous positions (AB) of all cancer cells in the biopsy; no loss or gain events have occurred at AB. LOH (B) is seen at the lower right corner with a high BAF, indicating that both alleles have been altered, i.e. point mutation and loss of wildtype. AB: diploid, one mutation and one wildtype. BB: diploid, loss of wildtype and gain of mutation. ABB: triploid, gain of mutation without loss of wildtype. Tumor front: contains the 3a-clone which has inherited the mutations of the 2b- and 1a-clones. Tumor center: contains 2a, 2b and 3a. Tumor back: contains 2a and 3c; 3c has inherited the mutations of 2b. Orange/blue highlighted LOH and BB seen for the mutations of 1a (BAF > 0.40) have occurred in one of the two clones. The highlighted orange/blue B’s indicate subclonality, grey B circles indicate events present in all cells. It is important to note that BBs originate from their closest B. It is not possible to determine in which clone these copy events have occurred (2a or 3c), as both clones occur with the same frequency, so their mutations are clustered together. Lymph node: contains 3b and 4a. The mutations of 3b and 4a are biopsy specific.

**Figure 3:**
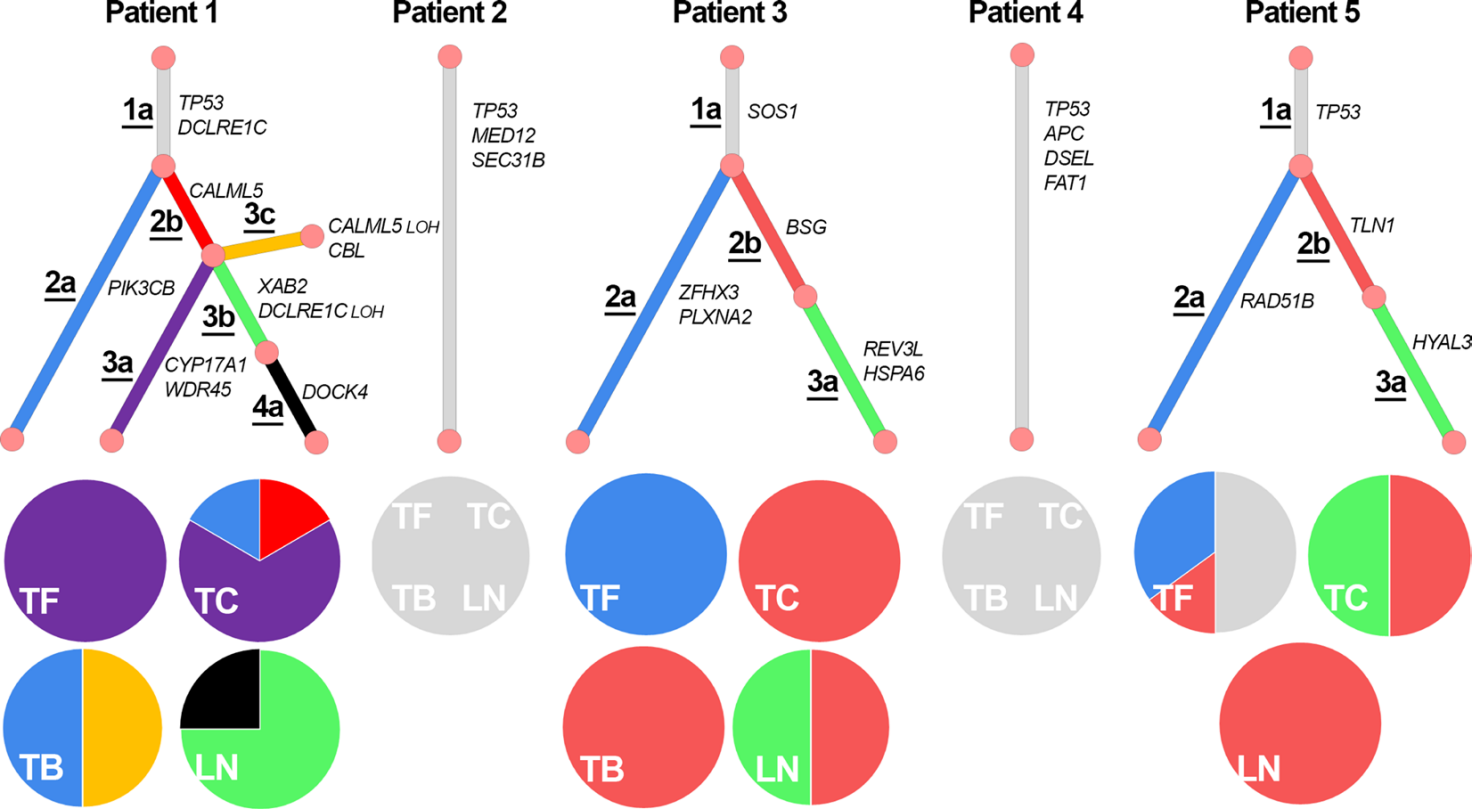
Phylogenetic trees for all patients Each pie chart represents a biopsy; they indicate the distribution of clones in each biopsy as the ratio between BAF(AB_subclone_) and BAF(AB_all_), please refer to Supplementary Note 1 for full details on construction of phylogenetic trees. Biopsy specific primary tumor mutations for patient 1 have been left out of the tree, since they either have a very low BAF or there are too few to base any conclusion on. Patient 5′s tumor back was left out, because it was evaluated not to contain any tumor by the pathologist, and the amount of mutations discovered are too few to base any conclusions on. There is no evidence of intra-tumor heterogeneity in patient 2 and 4. TF: Tumor front. TC: Tumor center. TB: Tumor back. LN: Lymph node.

In order to understand the subclonal evolution and progression of the cancers, we used copy number and mutational data to construct b-allele frequency vs copy number plots (Figure 2, Supplementary Figures 3–21). Based on these plots, we constructed a phylogenetic tree for each patient’s cancer (Figure 3) using the assumption that every clone inherits the ancestral clone’s somatic mutations, and each daughter subclone inherits their ancestor’s mutations. Analysis of the cancer in patient 1 indicates 7 clones. The 2b-clone of patient 1 has given rise to three different daughter clones, and the cancers of patient 3 and 5 also exhibit numerous clones. New clones for patient 1 and 3 arise in the lymph node metastasis. In contrast, the cancers of patient 2 and 4 seem to be very homogenous as their biopsies share the same mutations with no evidence of intra-tumor heterogeneity. Additionally, we used the recently published tool BubbleTree [17] to run an independent heterogeneity analysis using copy number data from germline variants and somatic copy number aberrations obtained from the exome sequencing. The results verify our estimated tumor contents and the existence of the most prominent subclones we find, supporting our phylogenetic analysis.

### Candidate cancer drivers

In the challenging process of identifying driver mutations, we analyzed recurrently mutated genes, bi-allelic alterations in the same gene, mutation type, iCAGES [18] candidate driver mutations, copy number and survival (Supplementary Tables 3, 4, 5, 6, 7, 8, 9, 10, Supplementary Figure 2). Using this approach, we identified 21 genes as possible candidate cancer driver genes (Table 1), including 7 top candidate genes: *TP53*, *FAT1*, *DSEL*, *CALML5*, *DCLRE1C*, *MUC16* and *KBTBD8*. A few are already established as cancer related in the Catalogue Of Somatic Mutations In Cancer [19] or previous HNSCC studies, i.e. *TP53*, *FAT1* [20] and *MUC16*, while the other 4 could possibly be novel drivers:

**Table 1:** Candidate cancer driver genes

Gene	Recurrence	iCAGES	Loss-of-function	LOH	COSMIC (%)	HR (95% CI)
*TP53*	X	X	X	X	0.274	n.s.
*FAT1*	X		X	X	0.028	n.s.
*DSEL*		X	X	X	0.010	n.s.
*CALML5*		X	X*	X**	0.002	1.39 (1.03−1.89)
*DCLRE1C*			X	X	0.004	1.40 (1.04−1.89)
*MUC16*	X		X		0.077	1.82 (1.34−2.49)
*KBTBD8*			X	X	0.005	1.41 (1.02−1.96)
*APC*		X		X	0.113	n.s.
*BRCA1*		X		X	0.017	n.s.
*CBL*		X		X	0.016	n.s.
*CHD7*			X	X	0.022	n.s.
*CYP17A1*		X		X	0.004	n.s.
*DST*	X		X		0.016	n.s.
*HSPA6*		X	X		0.005	n.s.
*MYH14*	X		X		0.010	n.s.
*NUPL1*			X	X	0.004	n.s.
*PAFAH1B2*			X	X	0.000	n.s.
*PHIP*			X	X	0.011	n.s.
*SPRY2*		X		X	0.002	n.s.
*USP8*		X		X	0.007	n.s.
*WDR45*			X	X	0.004	n.s.

To qualify as a candidate, the gene must at least fulfill 2 of the 4 criteria: Recurrence, iCAGES, Loss-of-function and LOH. The top 7 candidate genes have fulfilled 3 criteria and/or have a significant hazard ratio; these genes will be mentioned in the main text. Recurrence: is the gene recurrently mutated in our cohort. iCAGES: has the mutation been classified in iCAGES as a driver. Loss-of-function: is the mutation a frameshift indel, splice site or stopgain. LOH: has the gene undergone LOH. COSMIC: Percentage of gene mutated in the Catalogue of Somatic Mutations in Cancer. HR: Hazard ratio for loss of gene based on The Cancer Genome Atlas’s data from 522 HNSCC patients; *p*-value < 0.05 (Supplementary Figure 2). N.s.: not significant. X*: Missense mutation of *CALML5* resulted in a significant ion charge change from negative to positive (glutamic acid to lysine). X**: LOH of *CALML5* occurred independently in 2 distinct subclones.

*CALML5* not only gets mutated in patient 1 but also undergoes LOH, independently, in both the 3b- and 3c-clones. *DCLRE1C* also undergoes two alterations in patient 1, firstly an early stopgain mutation in the1a-clone thereafter LOH in the 3b-clone in the nodal metastasis; both *CALML5* and *DCLRE1C* are located on 10p. *DSEL* (synonym: *C18orf4*) and *KBTBD8* both have a loss of function point mutation and undergo loss of the other allele.

## DISCUSSION

Ultra-deep targeted sequencing enabled us to obtain the subclonal structure with a previously unseen high resolution, and it enabled us to determine complex copy number events based on the clustering of point mutations (see for example Figure 2). Approximately 80% of the possible variants were confirmed. We chose to use very loose criteria for the selection of possible variants for validation, which explains why the number is not higher. This was done to minimize false negative variants, but by using loose criteria, a higher number of false positive variants are selected for validation. However, these false positive variants were consequently not validated because of the high coverage ultra-deep sequencing allows for. Our analysis of intra-tumor heterogeneity reveals a low number of prominent clones in each biopsy, from 1 to 3 clones. This approach does not reject the possibility that minor subclones could exist at a very low frequency, as a reflection of the dynamic process of de novo mutations and selection. Our method for sampling multiple biopsies per primary tumor was chosen because it is easy and practical for the surgeon, it is consistent between different tumor sizes as we can change the size of the biopsy, and most importantly, it does not hinder the pathologist’s clinical analysis which is vital for determining the best possible treatment.

Analysis of the metastatic evolution revealed that in patient 1, 3 and 5 we observe additional specific primary tumor mutations that are not present in the lymph nodes, furthermore, none of the lymph nodes contain any mutations that are specific to a single primary tumor location. This indicates that the metastatic potential is acquired early in the tumor evolution, because the primary tumor specific mutations were acquired after the cancer disseminated to the lymph node. In 3 of 5 cases, we did not observe new mutations in the metastasis which could indicate that no new mutations are needed for survival and colonization. The latter is supported by a recent HNSCC study of nodal metastasis that shows a low degree of metastasis specific mutations [11]. However, low tumor content in patient 2, 4 and 5′s lymph node metastases lowers the resolution and ability to identify unique metastasis specific mutations by whole-exome sequencing in these patients. The tumor contents are high enough to confirm prominent mutations shared with their corresponding primary tumors due to the use of ultra-deep sequencing, but the detection of low frequency subclonal mutations is hampered even with our approach’s high coverage. To avoid this limitation in future studies, we recommend using a different sampling method to increase the tumor content in biopsies obtained from lymph node metastasis. We suggest that during the clinical assessment of lymph nodes the pathologist should determine tumor content, and take one or more samples from high tumor content areas for later sequencing. The lymph node biopsy should not be taken during surgery, as it can be hard to differentiate between cancerous and fibrous tissue.

Two types of mutations can exist in the lymph node, the first type of mutations originate from a clone in the primary tumor. These mutations are observed in all cancer cells in the lymph node. The second type of mutations are metastasis specific mutations only seen in the lymph node, either in all cancer cells or in a subclone. However, it is still possible that the unique mutations seen in all cancer cells, in the lymph node, could exist in their corresponding primary tumors, in a part we have not sampled or with a frequency below the detection limit.

Parallel evolution [21] is observed in the primary tumors of patient 1, 3 and 5; however, metastatic evolution is different in each case. For patient 1, parallel metastasis is observed as multiple new subclones arise in the primary tumor as well as in the node. Parallel metastasis is also seen in patient 3, as a new clone arises in the primary tumor and in the node. In patient 5 there are no signs of new clones in the metastasis. However, the primary tumor has evolved a new daughter clone originating from the same ancestral clone as the metastasis originates from. No sign of polyclonal seeding is evident, as all the nodal metastases seem to be monoclonally seeded from their corresponding primary tumor. Our observations indicate that OSCC is different from other cancer types like breast cancer, where the metastases seem to originate from advanced subclones in the primary tumor [22], and prostate cancer where polyclonal seeding has been observed [23].

Examining the HPV p16 status of the primary tumors reveals that patient 5 is the only positive case; however, HPV infection does not seem to be the driving factor for carcinogenesis in this patient. HPV-negative tumors exhibit higher mutation rates than HPV-positive HNSCC tumors [1–3, 10]. In this case, 116 mutations were identified in patient 5 compared to the average of 111 mutations in the remaining 4 HPV-negative cases. Additionally, the mutational profile of HPV-positive and HPV-negative are different with only a few overlapping gene mutations [10], while in contrast, we observe 11 recurrent gene mutations in patient 5 compared to the average 8 in the HPV-negative tumors (Supplementary Table 10). *TP53* is a common mutated gene in HPV-negative HNSCC tumors in contrast to HPV-positive tumors [1–3, 8, 24], a gene also seen mutated in patient 5 which further supports the notion that HPV infection does not seem be the driving factor for carcinogenesis.

The higher nonsynonymous to synonymous ratio than the expected 2:1 reflects competitive advantage with positive selection of nonsynonymous mutations. This suggests a high number of cancer drivers in our population. Our approach to identify candidate cancer drivers revealed 4 possible novel candidate driver genes in OSCC: Firstly, *CALML5* which encodes a skin-specific calcium-binding protein [25, 26] that is involved in epidermal differentiation [27]. K63-linked ubiquitination of the CALML5-protein in premenopausal breast cancer patients is reported to be strongly implicated in carcinogenesis [26].

*DCLRE1C* which encodes the Artemis protein involved in DNA repair [28]. Cells with an Artemis protein deficiency are more sensitive to radiation [29], as they show a higher incidence of chromosome breaks following irradiation [30], which could be of therapeutic interest for tumors with loss-of-function *DCLER1C* mutations.

*DSEL* (synonym: *C18orf4*) which encodes the dermatan sulfate epimerase-like protein that shows a significant homology with DSEP [31, 32] (synonym: SART-2), a squamous cell carcinoma antigen that can induce HLA-24-restricted and tumor-specific cytotoxic T-lymphocytes [33]. Loss of *DSEL* may have weakened the immune system’s response to the cancer.

Lastly, *KBTBD8* which encodes a protein that recently was found co-localizing with α-tubulin on the spindle apparatus of mitotic cells suggesting a role in cell proliferation. However, further studies are needed to investigate this assumption [34]. The 4 identified novel candidate genes need to be investigated further in functional studies, before the certainty of their involvement in carcinogenesis and metastasis can be established.

Our results demonstrate that ultra-deep sequencing provides unseen high resolution enabling clear detection of subclonal structure. Primary treatment of OSCC is usually surgery but adjuvant radiotherapy can be applied [35]. Chemotherapy is used as a part of adjuvant treatment of late stage cancer, recurrence and metastasis [35], but not all patients can handle such a regime due to side effects and comorbidities. Future targeted medical treatments are needed to increase survival and reduce side effects, enabling more vulnerable patients to receive treatment.

In cancer research, two clinical challenges regarding heterogeneity are currently being vigorously discussed: Is a single biopsy representative of the primary tumor’s mutational profile, and is a single biopsy taken from the primary tumor representative of metastasis. Our results indicate that OSCC is a cancer with many driver mutations, with a high degree of inter-patient heterogeneity but a low degree of intra-tumor heterogeneity. All biopsies from each patient share the majority of mutations and only a low number of prominent subclones exist. In several cases we find mutations in the primary tumor that are not present in the lymph node metastasis, which indicates that the metastatic potential, in our population, is acquired early in tumor evolution. It might just be a matter of time before metastasis occurs.

## MATERIALS AND METHODS

### Patient selection and sampling

Ethics approval was obtained from The Regional Scientific Ethical Committees for Southern Denmark and informed consent were acquired from the 5 patients that were included in this study. The study was carried out in accordance with the approved guidelines. All patients were characterized as having stage III or IV oral cavity carcinomas with cervical lymph node involvement. All patients were of Caucasian descent. Average age of the group was 56.2 years; 3 out of 5 had a history of smoking, and all patients had in various degrees consumed alcohol. Two patients were characterized as heavy drinkers. The tumors were tested for p16 overexpression which is used as a prognostic factor for HPV infection. None of the patients had distant metastasis, and no patient had received treatment for their condition prior to their operation. Patient characteristics are outlined in Supplementary Table 1. The operations took place at the Department of Plastic Surgery, Odense University Hospital, Denmark, which is the center for surgical treatment of oral cavity cancer for the Region of Southern Denmark’s 1.2 million inhabitants. From each patient, 5 samples were collected. One blood sample consisting of 10 mL of venous blood was drawn into a heparinized collection tube. Primary site tumor biopsies were taken from 3 different sites of the resected tumor: front, center and back. Lastly, 1 lymph node with signs of metastasis that was extracted during the neck dissection was collected. All samples were freshly frozen and stored at −80° Celsius for later use.

### Pathology

The 20 tissue biopsies were evaluated by a pathologist to confirm the presence of squamous tumor cells. One biopsy, tumor back from patient 5, was evaluated not to contain any tumor tissue; the remaining primary tumor and lymph node biopsies contained between 20 to 80% tumor. To minimize the presence of normal tissue in the lymph nodes, the pathologist marked the areas that contained tumor before being macroscopically dissected.

### DNA extraction

DNA from the 10 mL whole blood was extracted using the Gentra PureGene Blood kit (Qiagen) following the instructions provided by the manufacturer. DNA was extracted from approximately 30 mg of primary tumor and lymph node biopsies using the AllPrep DNA/RNA Mini kit (Qiagen).

### Exome sequencing and validation

DNA extracted from the samples were subjected to sample preparation and exome capture by hybridization using TruSeq Exome Enrichment kit (Illumina) following the standard protocol provided by the manufacturer. Sequencing was carried out on the Illumina HiSeq1500 platform with paired-end 2 × 100 base-pair reads. The filtered variants were validated by enriching the samples using Agilent SureSelect XT, and then sequenced on the same platform with a much higher coverage. Validation of variant positions was performed in all samples from all patients and not just in the samples where they were detected. Exome sequencing results of tumor center and back from patient 3 were not retrieved due to technical problems. However, both underwent validation, but it is only the mutations in tumor front and the lymph node that have been validated. No biopsy specific mutations have been validated in these biopsies.

### B-allele frequency

The B-allele frequency (BAF) represents the fraction of alternative reads (B-allele) in the tumor biopsy related to the sum of the reference reads (A-allele) and alternative reads. BAF is calculated as

$$\text{BAF}=\frac{B-\text{allele reads}}{A+B-\text{allele reads}}$$

BAF is calculated for somatic point mutations, but also for germline variants for use in copy number estimates.

### Bioinformatics

Raw reads were aligned to the hg19 reference genome using Novoalign v. 3.01 (Novocraft) and processed according to Genome Analysis ToolKit Best Practice pipeline v. 2.7 (Broad Institute), including duplicate removal, indel realignment and base quality score recalibration [36, 37]. Calling of variants was performed using Varscan v. 2.3.4 [38], and Annovar (2013Aug23) [39] was used for annotation of variants. dbSNP build 138 [40] was used for filtering out known germline mutations. Only bases with a quality score of at least Q20 (corresponding to an error rate of 1:100) were considered. We used the following criteria to identify somatic mutations derived from the exome data:

1. A variant should only be called if it had a BAF of at least 5% and had ≥ 3 alternative reads in one of the samples besides blood.

2. To ensure that the B-allele was not a germline variant, the blood sample should at least have 10× coverage at the same position and have 0 alternative reads.

The filtered variants were validated using ultra-deep sequencing. Before analyzing the validated data, we used the following criteria to ensure a reliable analysis:

1. A variant should only be called if it had a BAF of at least 3% and had ≥ 10 alternative reads in one of the samples besides blood.

2. To ensure that the B-allele was not a germline variant, the blood sample should at least have 50× coverage at the same position and have a BAF ≤ 1%.

As stated, if a variant has a BAF of at least 3% and at least 10 alternative reads in one of the tissue biopsies, the variant will be called. This consequently means that we are more certain of the existence of the same variant in the other biopsies, even if BAF is under 3%; however, alternative reads should be sufficiently high enough to avoid false positive variants.

### BAF vs copy number plots

Copy number estimates were generated using ngCGH with a window size of 10,000 reads (https://github.com/seandavi/ngCGH). LogR was defined as the Log2 copy number ratio between tumor and matched normal sample. Construction of the B-allele frequency vs copy number plots was performed by first finding the corresponding LogR value of each somatic mutation. This was done by creating a script in R which would search for each position in the raw copy number files obtained from ultra-deep sequencing. The values present in these files are representative for a range of positions; they are not values for each specific position. Essentially, the LogR value retrieved would be a mean estimate for the neighboring single nucleotide polymorphisms (SNPs), as the copy number values are based on SNPs and not on somatic mutations. For each patient, the values were plotted against each other, and the mutations were color coded during the analysis. Mutations were colored depending on how many biopsies they appeared in; if they appeared in all the biopsies they were not color coded (Supplementary Figures 3–21).

### BubbleTree

BubbleTree, a recently published method developed for aneuploidy and clonal visualization was used to run an independent heterogeneity analysis based on the BAF and LogR values of germline variants obtained from the exome sequencing. The copy number data were first segmented by the DNACopy package [41].

### Phylogenetic trees

The phylogenetic trees are based on the BAF vs copy number plots (Supplementary Figures 3–21) and the mutational data (Supplementary Tables 3, 4, 5, 6, 7). Detecting possible subclones was done by visual interpretation of the plots (Supplementary Figure 22). Please refer to Supplementary Note 1 for full details.

### iCAGES

The iCAGES tool was used to identify driver mutations based on substitutional data. The tool includes 3 layers of analysis. First layer integrates structural variations from coding, non-coding and to infer driver variants. The second layer identifies driver genes, by using information from the first layer and prior biological knowledge on gene-gene and gene-phenotype networks. Third layer prioritizes drug therapy based on the identified potential driver genes. However, iCAGES cannot, at this moment, identify driver mutations which are indels or splice site mutations.

### TCGA data retrieval

Data available on 522 HNSCC patients were retrieved from of The Cancer Genome Atlas (TCGA) Research Network (http://cancergenome.nih.gov). The dataset (TCGA_HNSC_gistic2thd) was extracted through the UCSC Cancer Genomics Browser (https://genome-cancer.ucsc.edu) on the 20th of October, 2015. TCGA’s datasets include curated survival data which we use for survival analysis.

### Availability of data and material

The raw next-generation sequencing datasets generated during the current study are not publicly available due to it being against Danish legislation. However, interested parties will be able to obtain the data after consulting the Danish Data Protection Agency and The Regional Scientific Ethical Committees for Southern Denmark following a request to the corresponding author.

All other data analysed during this study are included in this published article and its Supplementary Information files.

## SUPPLEMENTARY MATERIALS FIGURES AND TABLES


