# Supplementary material for: The subclonal structure and genomic evolution of oral squamous cell carcinoma revealed by ultra-deep sequencing
Source: Oncotarget. 2017 Feb 2;8(10):16571–80. doi: 10.18632/oncotarget.15014 (PMC5369985; doi:10.18632/oncotarget.15014)
Supplement: Supplementary file 1 [file oncotarget-08-16571-s001.pdf]

The subclonal structure and genomic evolution of oral squamous cell carcinoma revealed by ultra-deep sequencing

Supplementary Materials

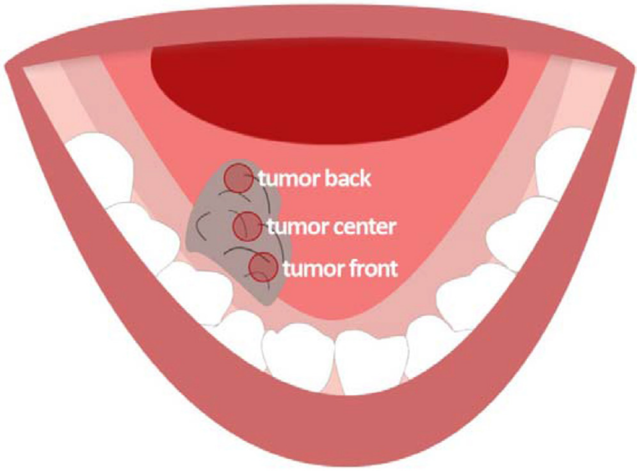

Supplementary Figure 1: Graphical illustration of primary tumor sampling.

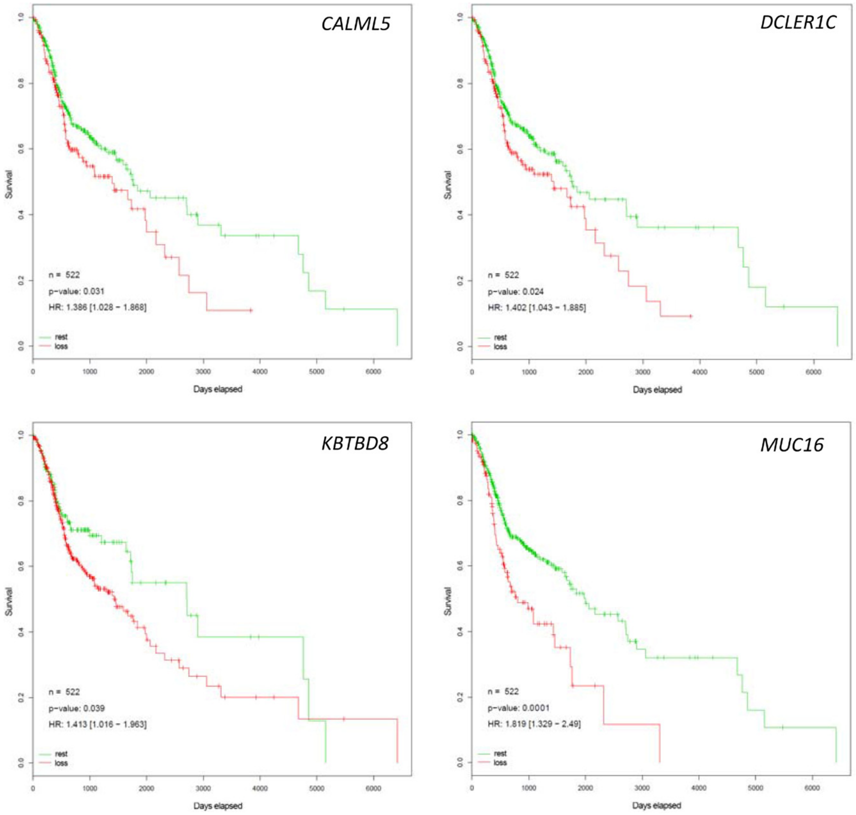

Supplementary Figure 2: Kaplan-Meier plots for loss compared to no loss.

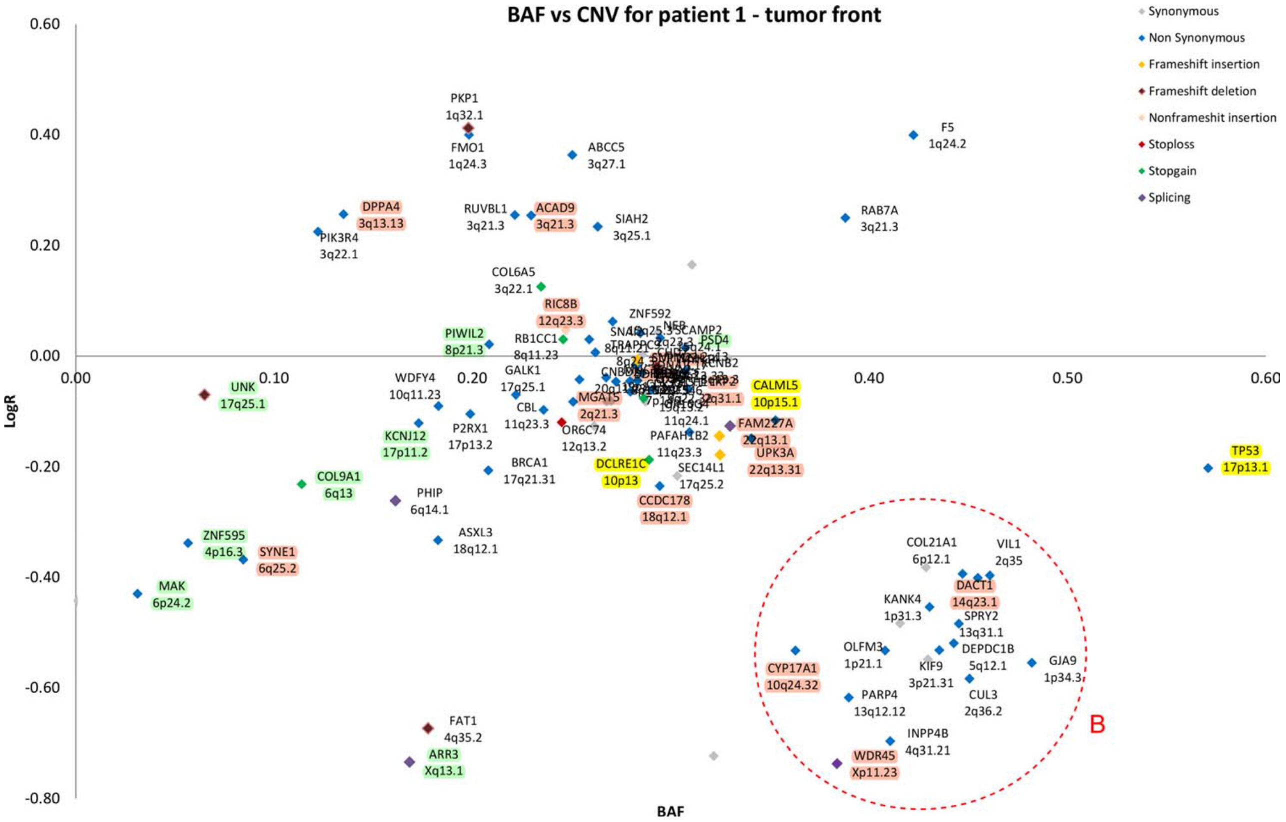

Supplementary Figure 3:

- Green highlighted mutations are unique for that specific biopsy.
- Red highlighted mutations are shared by tumor front and tumor center.
- Blue highlighted mutations are shared by tumor center and tumor back.
- Yellow highlighted mutations are mentioned in the article.
- B: Loss of Heterozygosity. AB: diploid, 1 wildtype, 1 mutant allele. ABB: triploid, 1 wildtype, 2 mutant alleles. BB: 0 wildtype, 2 mutant alleles.

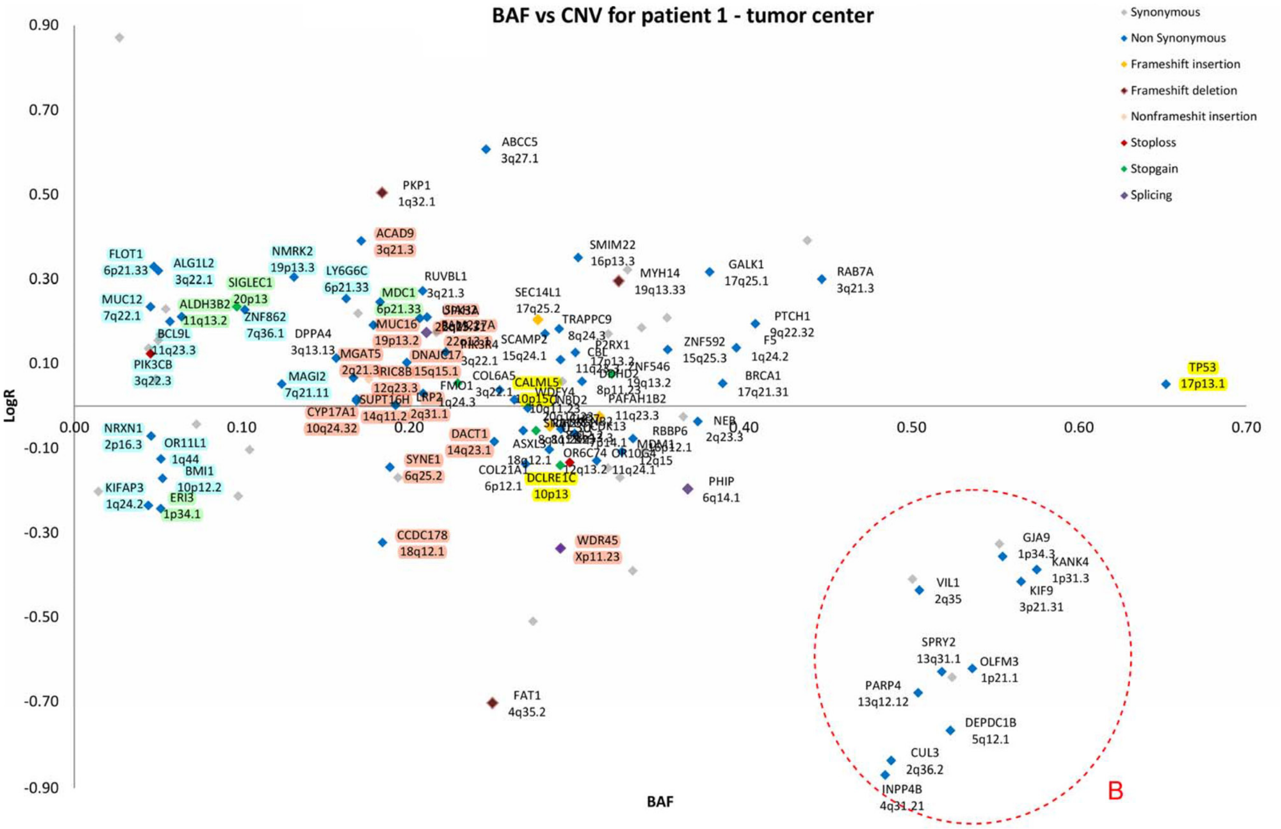

Supplementary Figure 4:

- Green highlighted mutations are unique for that specific biopsy.
- Red highlighted mutations are shared by tumor front and tumor center.
- Blue highlighted mutations are shared by tumor center and tumor back.
- Yellow highlighted mutations are mentioned in the article.
- B: Loss of Heterozygosity. AB: diploid, 1 wildtype, 1 mutant allele. ABB: triploid, 1 wildtype, 2 mutant alleles. BB: 0 wildtype, 2 mutant alleles.

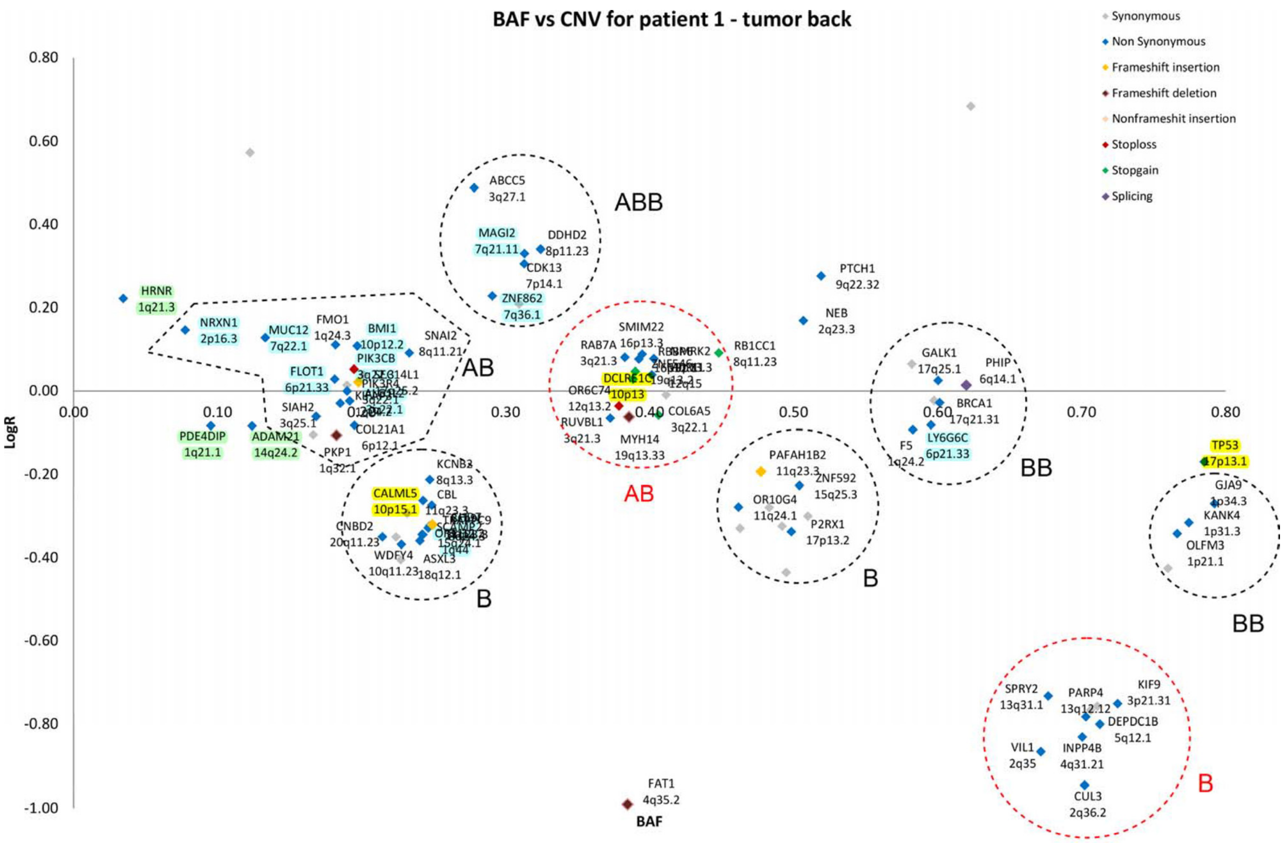

Supplementary Figure 5:

- Green highlighted mutations are unique for that specific biopsy.
- Red highlighted mutations are shared by tumor front and tumor center.
- Blue highlighted mutations are shared by tumor center and tumor back.
- Yellow highlighted mutations are mentioned in the article.
- B: Loss of Heterozygosity. AB: diploid, 1 wildtype, 1 mutant allele. ABB: triploid, 1 wildtype, 2 mutant alleles. BB: 0 wildtype, 2 mutant alleles.

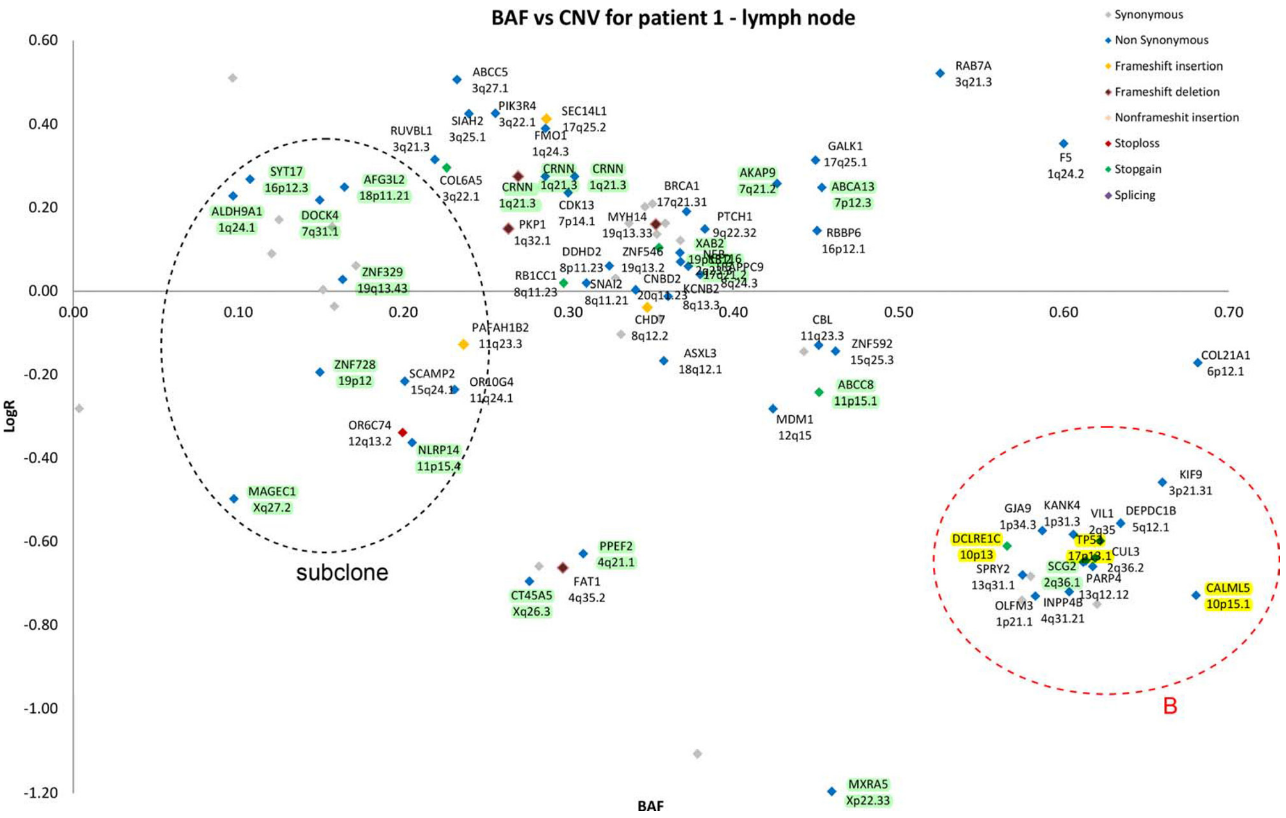

Supplementary Figure 6:

- Green highlighted mutations are unique for that specific biopsy.
- Red highlighted mutations are shared by tumor front and tumor center.
- Blue highlighted mutations are shared by tumor center and tumor back.
- Yellow highlighted mutations are mentioned in the article.
- B: Loss of Heterozygosity. AB: diploid, 1 wildtype, 1 mutant allele. ABB: triploid, 1 wildtype, 2 mutant alleles. BB: 0 wildtype, 2 mutant alleles.

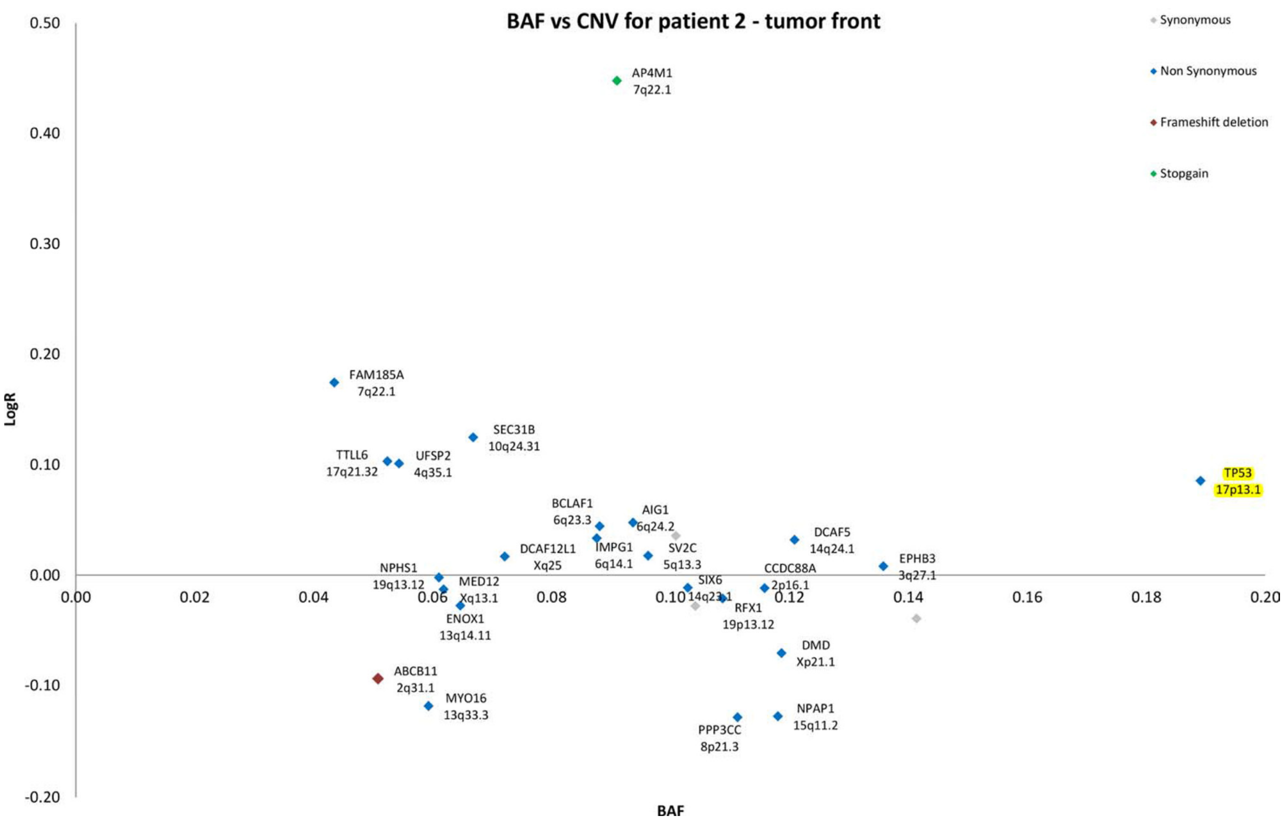

Supplementary Figure 7:

■ Yellow highlighted mutations are mentioned in the article.

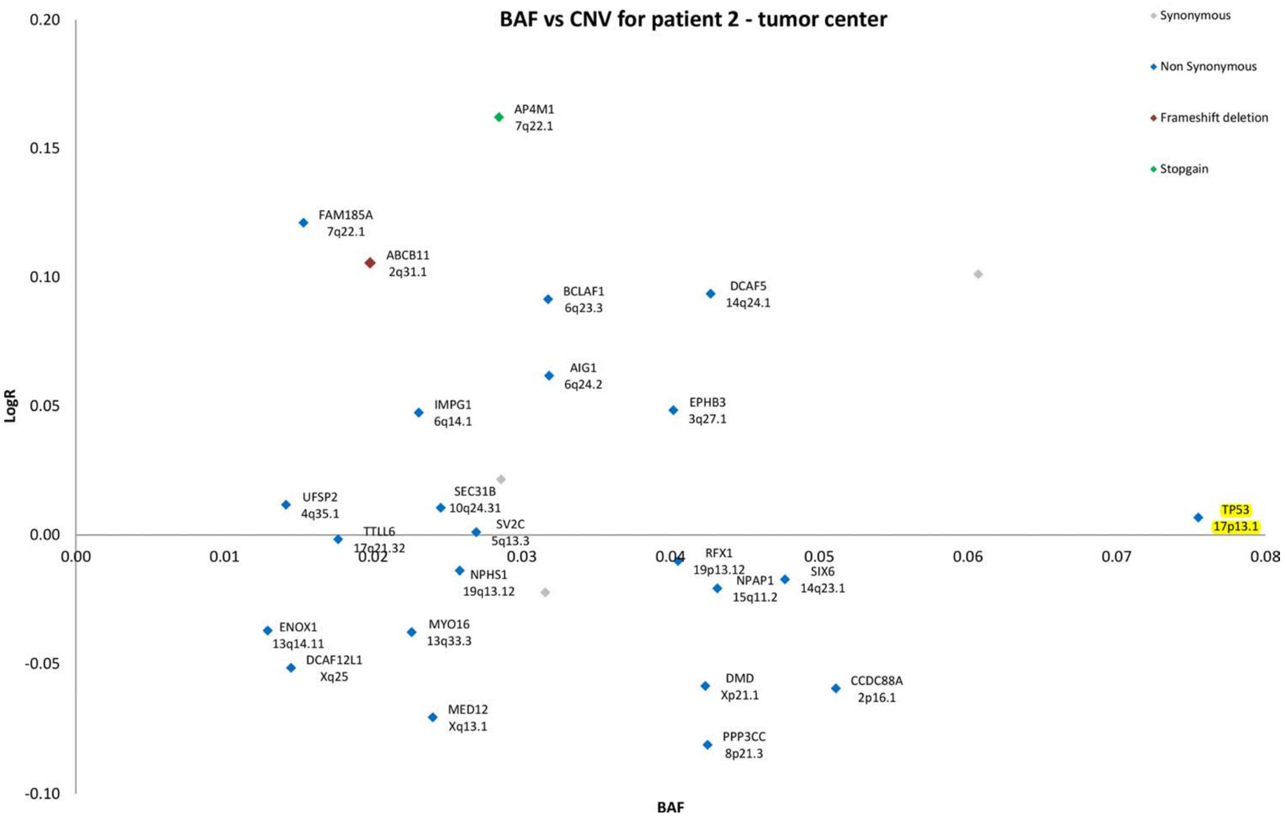

Supplementary Figure 8:

■ Yellow highlighted mutations are mentioned in the article.

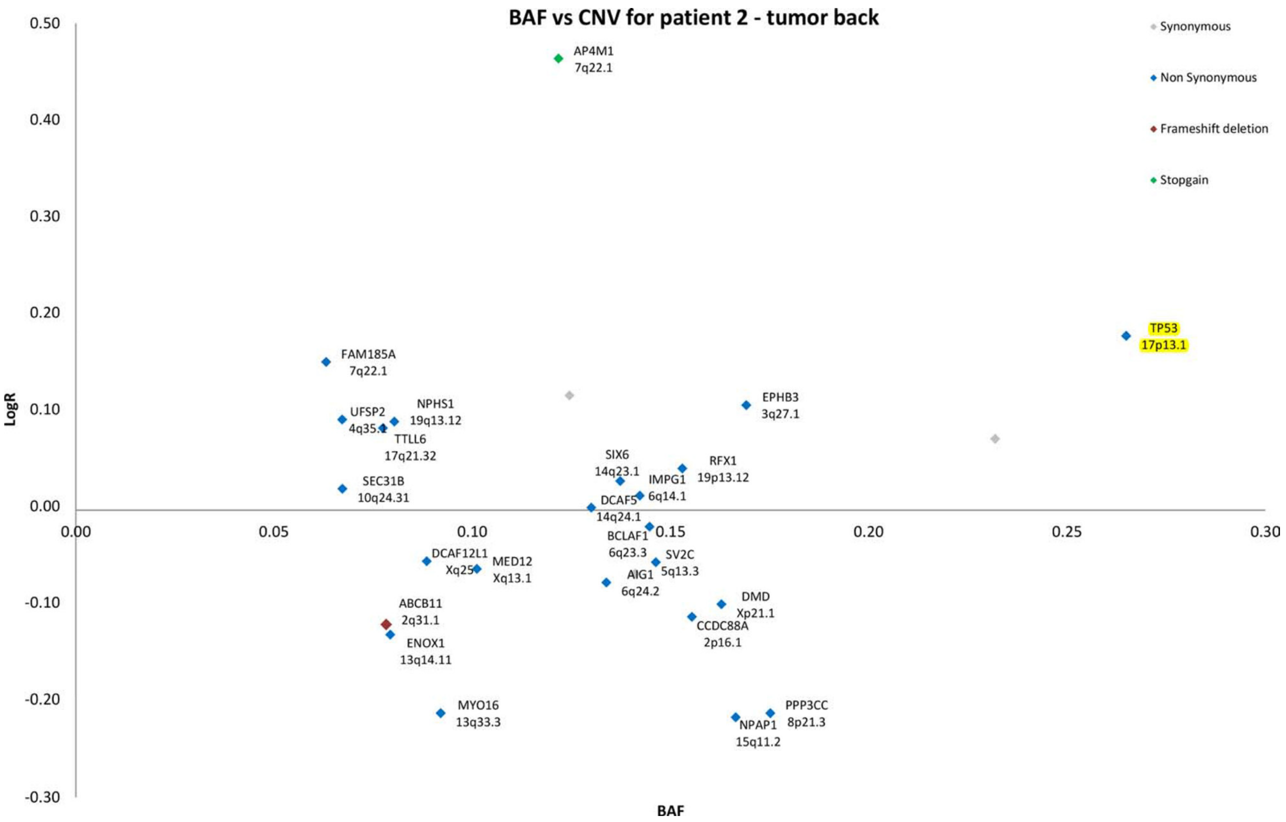

Supplementary Figure 9:

■ Yellow highlighted mutations are mentioned in the article.

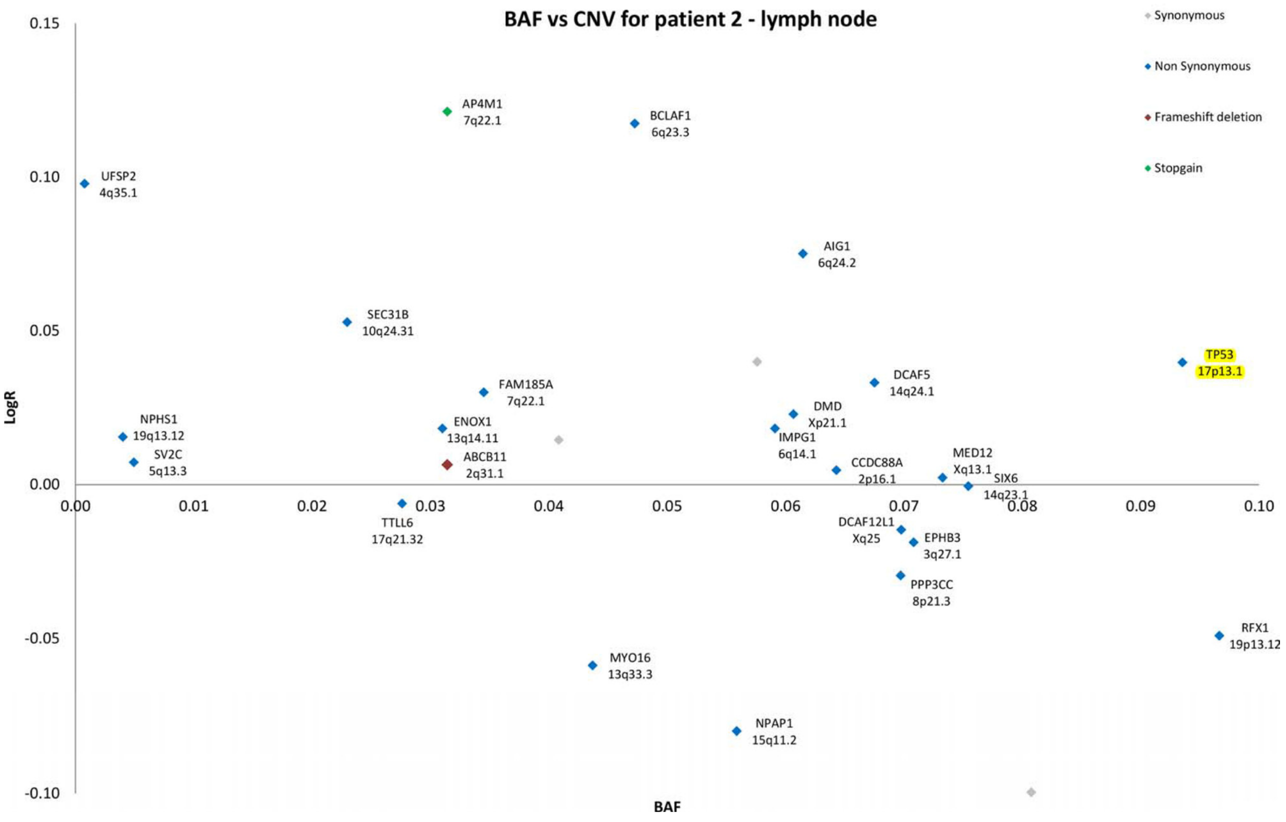

Supplementary Figure 10:

■ Yellow highlighted mutations are mentioned in the article.

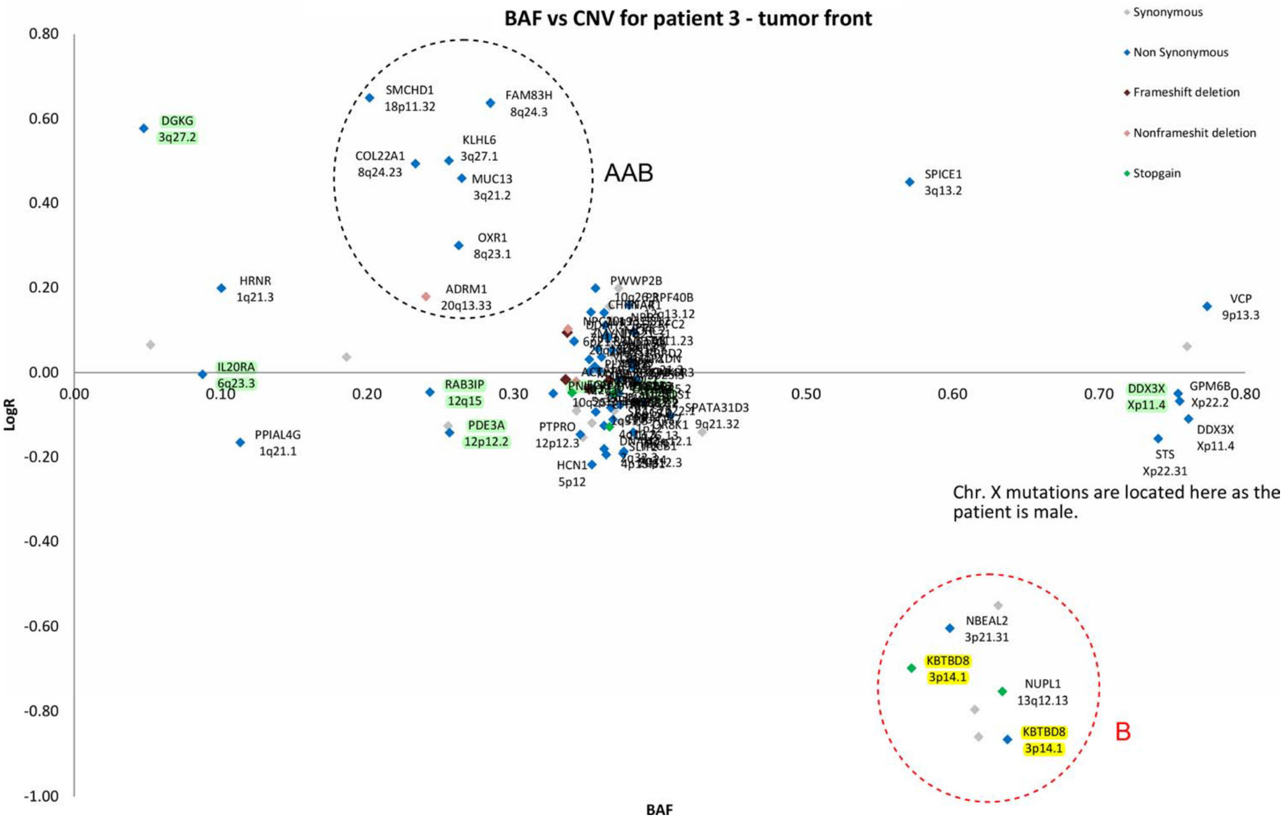

Supplementary Figure 11:

- Green highlighted mutations are unique for that specific biopsy.
- Pink highlighted mutations are shared by tumor center, back and lymph node.
- Yellow highlighted mutations are mentioned in the article.
- B: Loss of Heterozygosity. AAB: triploid, 2 wildtype, 1 mutant allele.

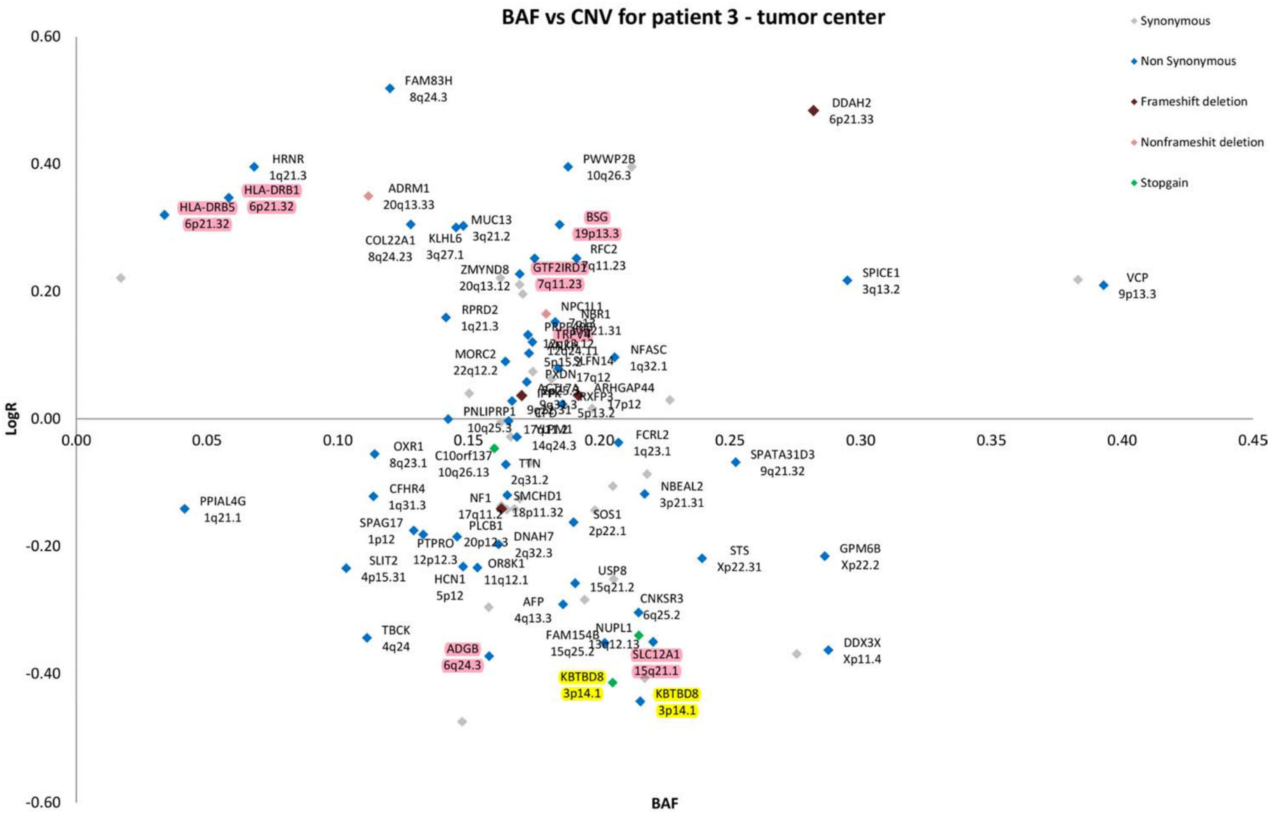

Supplementary Figure 12:

- Green highlighted mutations are unique for that specific biopsy.
- Pink highlighted mutations are shared by tumor center, back and lymph node.
- Yellow highlighted mutations are mentioned in the article.
- B: Loss of Heterozygosity. AAB: triploid, 2 wildtype, 1 mutant allele.

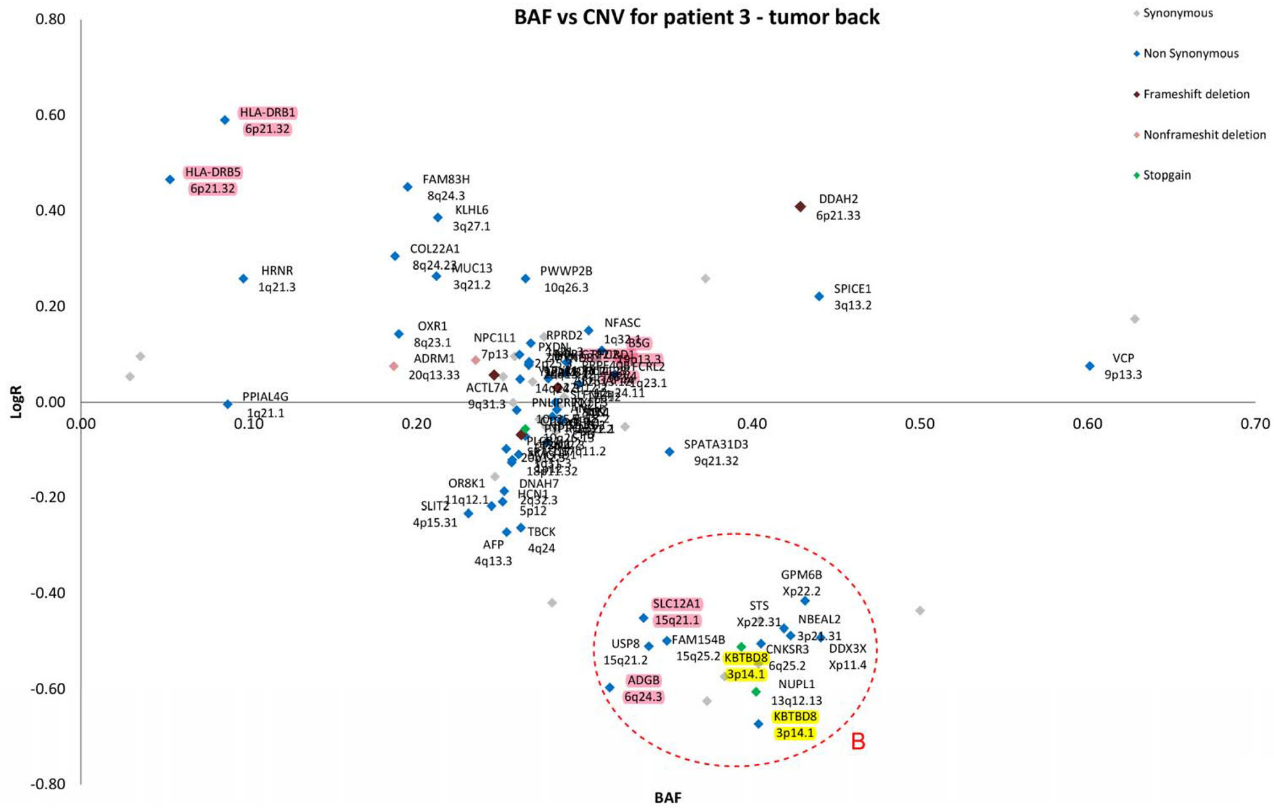

Supplementary Figure 13:

- Green highlighted mutations are unique for that specific biopsy.
- Pink highlighted mutations are shared by tumor center, back and lymph node.
- Yellow highlighted mutations are mentioned in the article.
- B: Loss of Heterozygosity. AAB: triploid, 2 wildtype, 1 mutant allele.

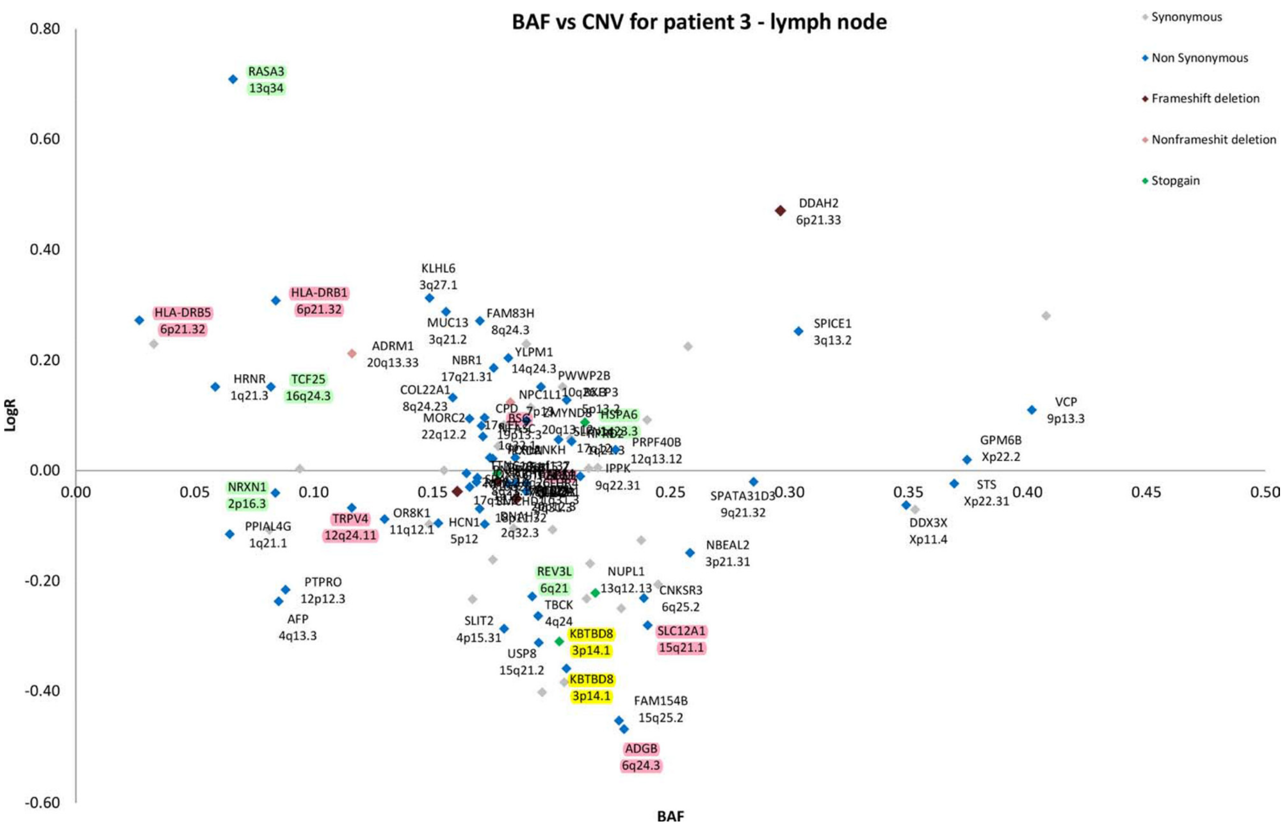

Supplementary Figure 14:

- Green highlighted mutations are unique for that specific biopsy.
- Pink highlighted mutations are shared by tumor center, back and lymph node.
- Yellow highlighted mutations are mentioned in the article.
- B: Loss of Heterozygosity. AAB: triploid, 2 wildtype, 1 mutant allele.

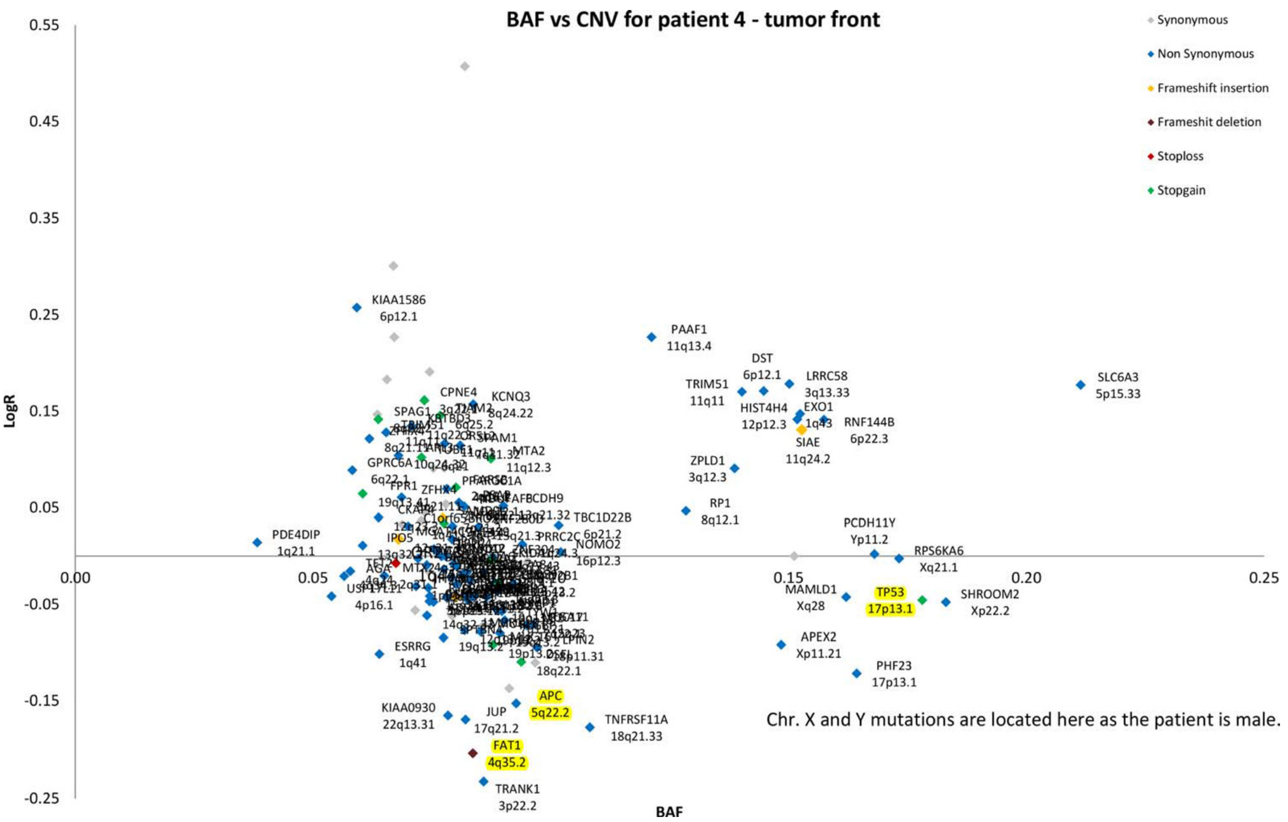

Supplementary Figure 15:

- Yellow highlighted mutations are mentioned in the article.
- B: Loss of Heterozygosity. AAB: triploid, 2 wildtype, 1 mutant allele. ABB: triploid, 1 wildtype, 2 mutant alleles.

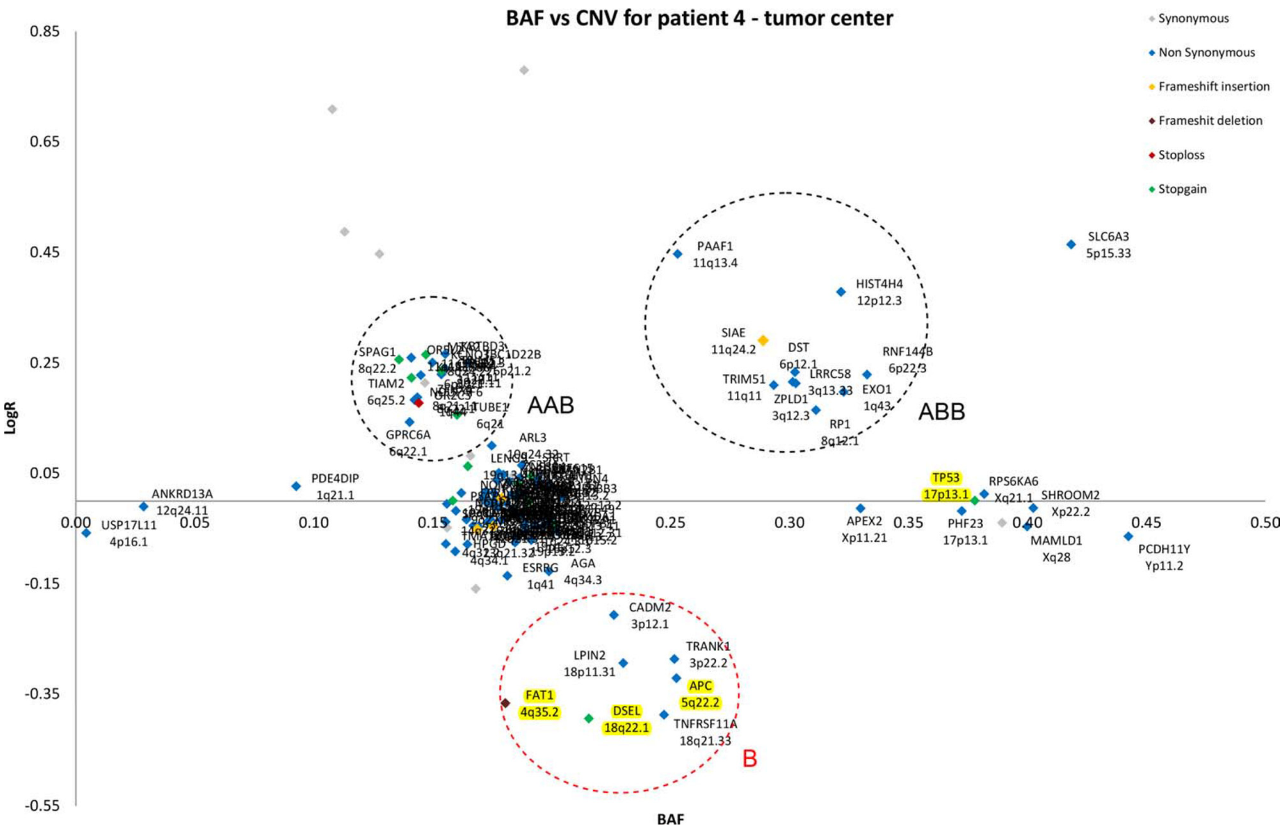

Supplementary Figure 16:

- Yellow highlighted mutations are mentioned in the article.
- B: Loss of Heterozygosity. AAB: triploid, 2 wildtype, 1 mutant allele. ABB: triploid, 1 wildtype, 2 mutant alleles.

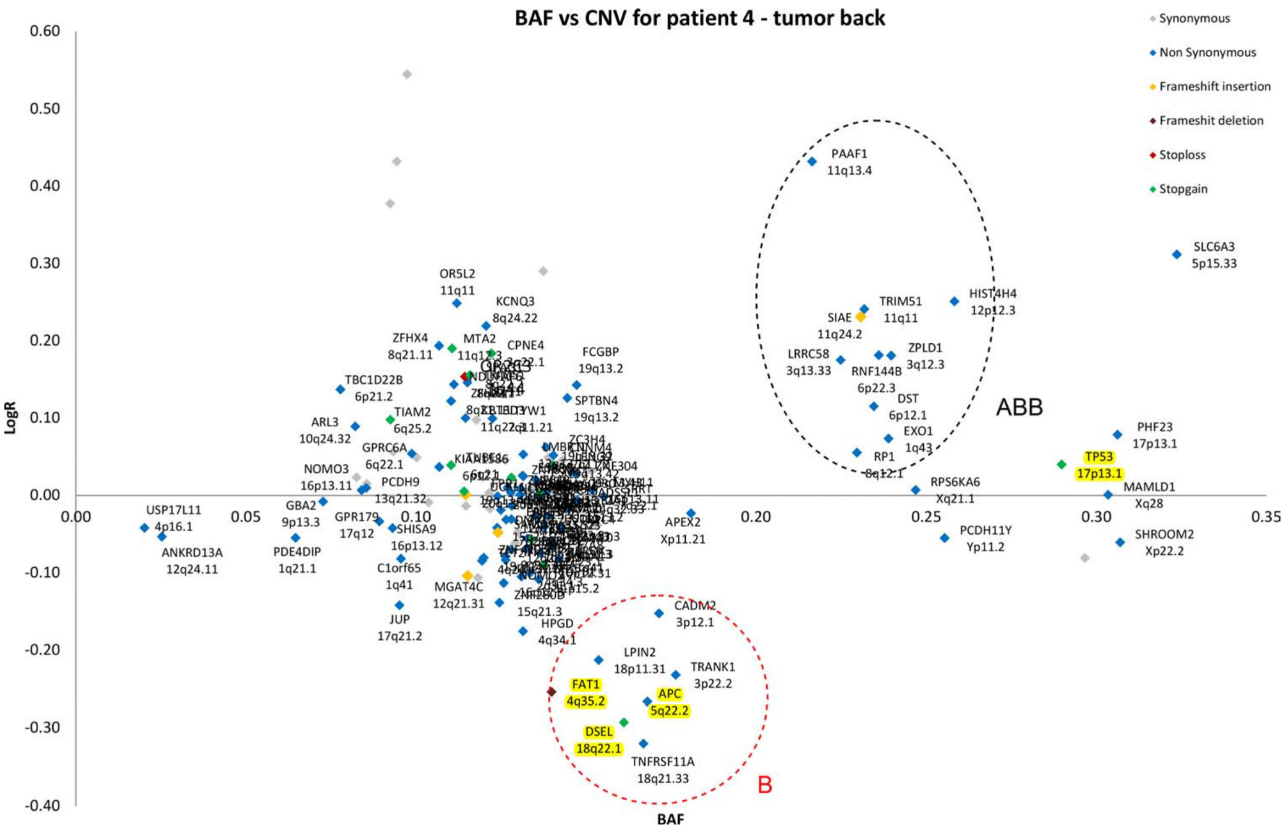

Supplementary Figure 17:

- Yellow highlighted mutations are mentioned in the article.
- B: Loss of Heterozygosity. AAB: triploid, 2 wildtype, 1 mutant allele. ABB: triploid, 1 wildtype, 2 mutant alleles.

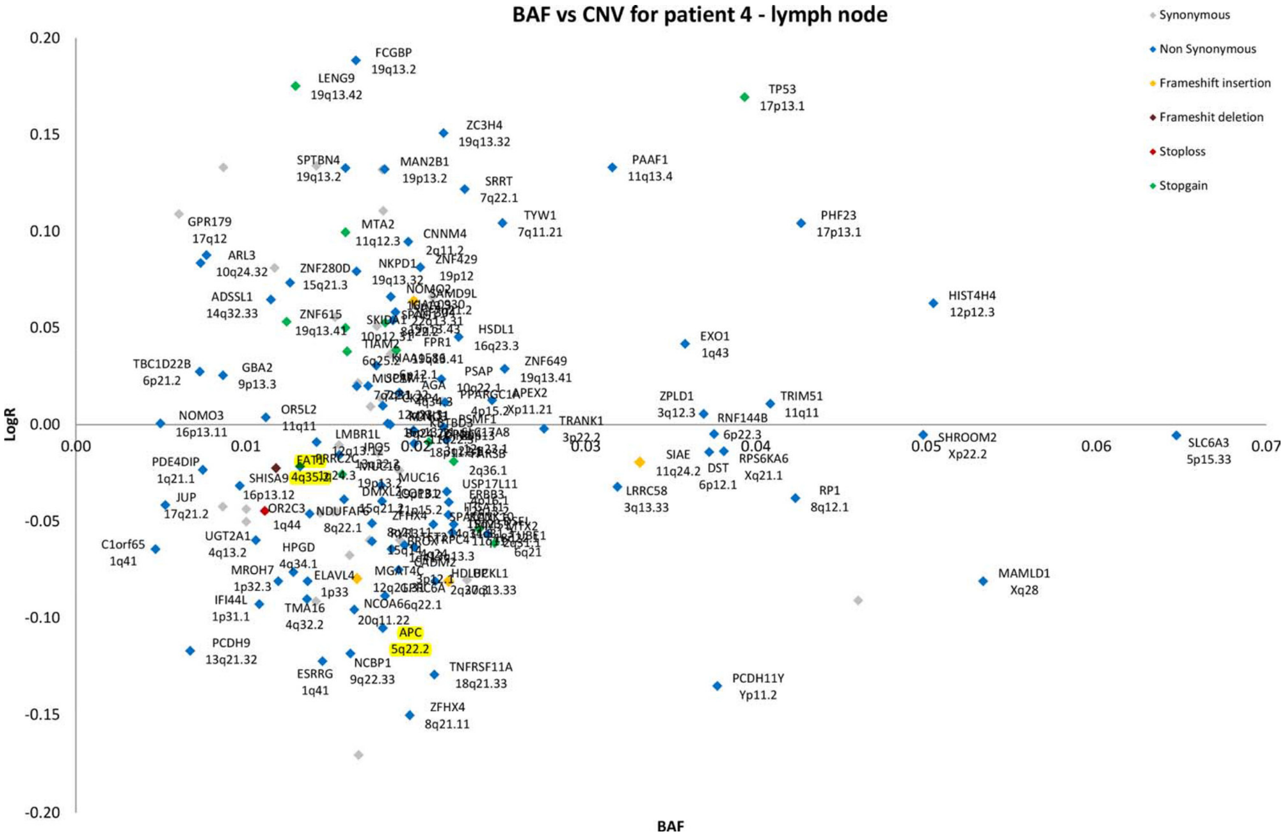

Supplementary Figure 18:

- Yellow highlighted mutations are mentioned in the article.
- B: Loss of Heterozygosity. AAB: triploid, 2 wildtype, 1 mutant allele. ABB: triploid, 1 wildtype, 2 mutant alleles.

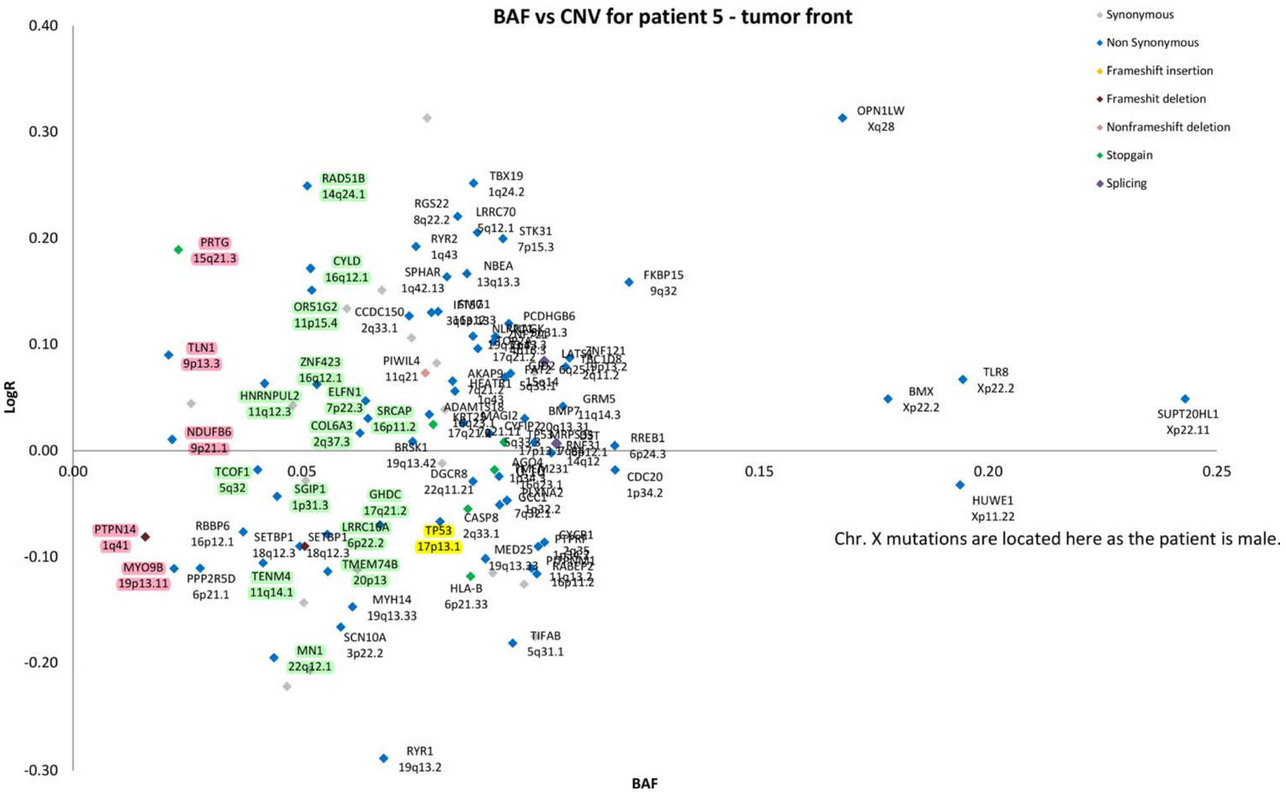

Supplementary Figure 19:

- ■ Green highlighted mutations are unique for that specific biopsy.
- ■ Pink highlighted mutations are shared by tumor front, center and lymph node.
- ■ Yellow highlighted mutations are mentioned in the article.

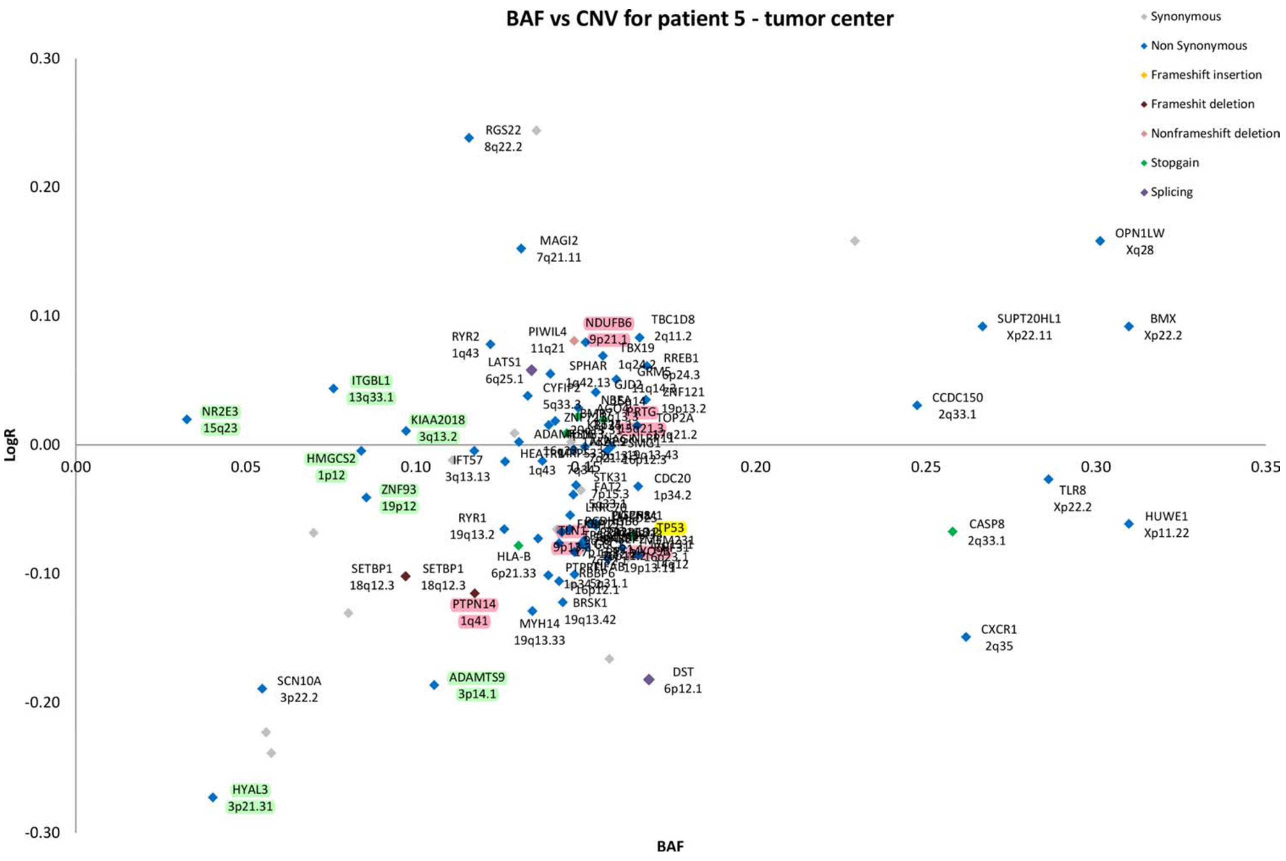
BAF vs CNV for patient 5 - tumor center
Synonymous
Non Synonymous
Frameshift insertion
Frameshit deletion
Nonframeshift deletion
Stopgain
Splicing
LogR
BAF
RGS22
8q22.2
MAGI2
7q21.11
OPN1LW
Xq28
SUPT20HL1
Xp22.11
BMX
Xp22.2
CCDC150
2q33.1
TLR8
Xp22.2
HUWE1
Xp11.22
CASP8
2q33.1
CXCR1
2q35
NR2E3
15q23
ITGBL1
13q33.1
KIAA2018
3q13.2
HMGCS2
1p12
ZNF93
19p12
SETBP1
18q12.3
PTPN14
1q41
SCN10A
3p22.2
ADAMTS9
3p14.1
HYAL3
3p21.31
DST
6p12.1
MYH14
19q13.33
BRSK1
19q13.42
HLA-B
6p21.33
RYR1
19q13.2
TP53
NDUFB6
9p21.1
TBC1D8
2q11.2
CDC20
1p34.2

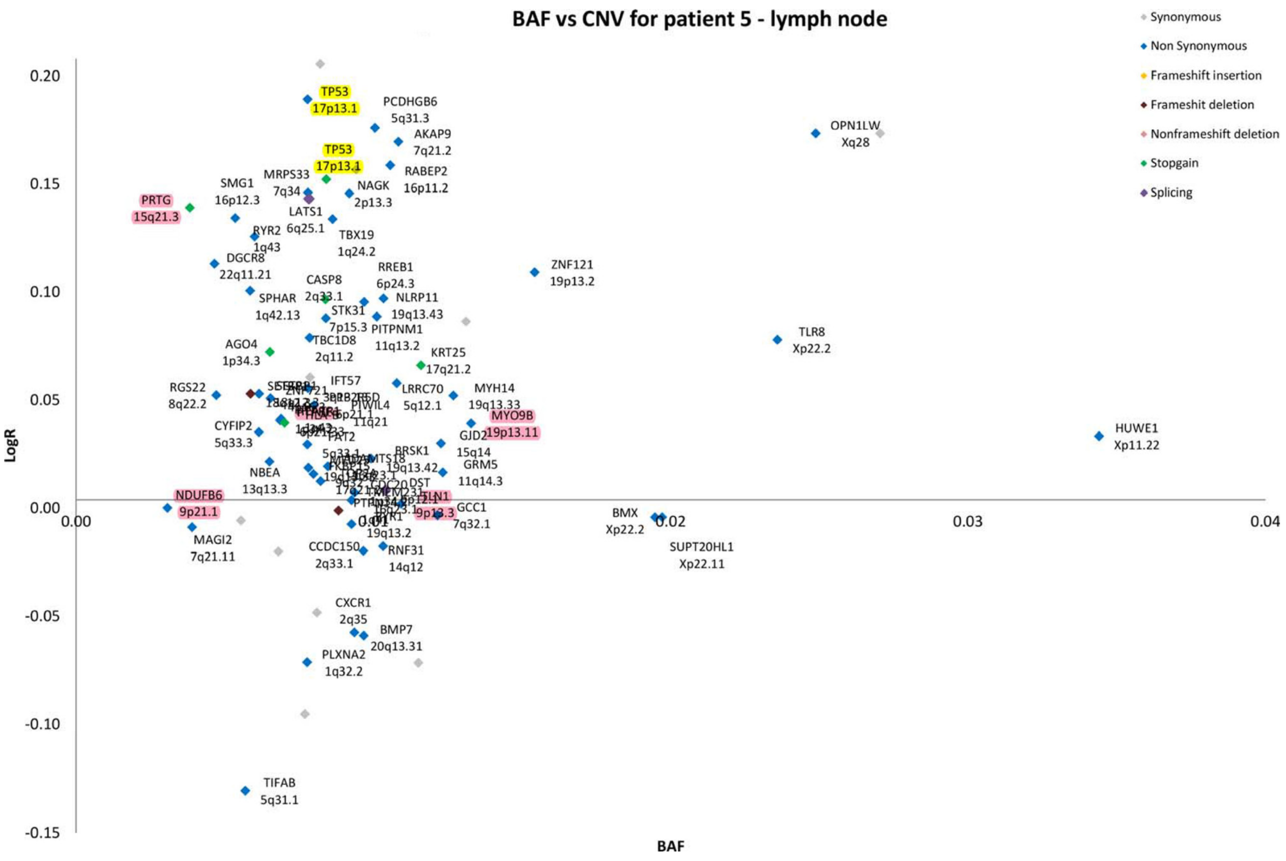

Supplementary Figure 21:

- Green highlighted mutations are unique for that specific biopsy.
- Pink highlighted mutations are shared by tumor front, center and lymph node.
- Yellow highlighted mutations are mentioned in the article.

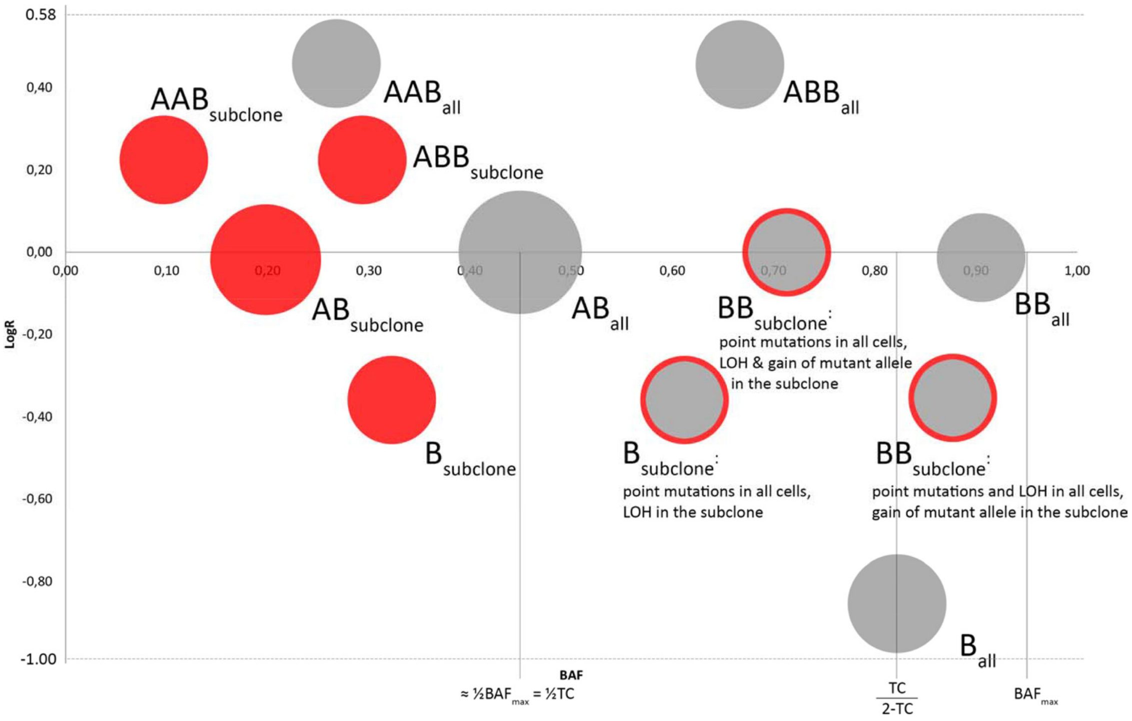

Supplementary Figure 22: Illustration of the visual interpretation method for the BAF vs copy number plot analysis in a situation with 90% tumor content and one subclone constituting 44 % of the tumor. LogR is defined as Log2 copy number ratio. BAF: B-allele frequency. TC: Tumor content. AB: diploid, 1 wildtype (A-allele), 1 mutant allele (B-allele). B: 1 mutant allele, Loss of Heterozygosity. AAB: triploid, 2 wildtype, 1 mutant allele. ABB: triploid, 1 wildtype, 2 mutant alleles. BB: 2 mutant alleles.

Supplementary Table 1: Patient characteristics

	Patient 1	Patient 2	Patient 3	Patient 4	Patient 5
**Age**	57	53	69	59	43
**Gender**	Female	Female	Male	Male	Male
**Site**	FOM	Glossus+FOM	FOM	Glossus	Buccal
**TNM**	T4N2M0	T4N1M0	T4N2M0	T3N2bM0	T1N1M0
**Smoking**	Smoker	Non-smoker	Smoker	Smoker	Non-smoker
**Alcohol**	Alcoholic	Occasional	Alcoholic	Occasional	Occasional
**HPV p16 Status**	Negative	Negative	Negative	Negative	Positive

Site: primary site of the tumor. FOM: Floor of mouth. TNM: Tumor-Node-Metastasis staging = T: tumor size and invasion N: regional lymph node, M: distant metastasis.

Supplementary Table 2: Stats for ontarget sequence

		Whole exome sequencing (62 Mb)			Validation		
	**Tissue**	**Duplication**	**Coverage**	**Bases > 20**	**Duplication**	**Coverage**	**Bases > 100**
Patient 1	**Blood**	14,6%	62,0	79,5 %	57,6 %	501,8	90,9 %
	**Lymph node**	5,0%	73,9	81,9 %	20,4 %	1386,0	98,9 %
	**Tumor back**	5,9%	100,7	83,4 %	20,7 %	2339,5	99,2 %
	**Tumor front**	5,7%	94,6	84,2 %	20,8 %	1431,6	99,0 %
	**Tumor center**	5,1%	83,0	83,2 %	20,9 %	2081,9	99,1 %
Patient 2	**Blood**	5,1 %	54,7	80,4 %	22,9 %	2456,1	99,0 %
	**Lymph node**	5,4 %	93,0	85,2 %	22,7 %	2222,0	99,0 %
	**Tumor back**	5,2 %	86,9	85,1 %	24,0 %	2134,9	99,0 %
	**Tumor front**	5,0 %	67,7	82,9 %	22,5 %	1405,5	98,6 %
	**Tumor center**	5,0 %	79,7	84,7 %	23,5 %	1667,7	98,8 %
Patient 3	**Blood**	7,8 %	105,8	84,3 %	21,9 %	1478,4	98,7 %
	**Lymph node**	8,0 %	136,0	85,8 %	21,2 %	1486,5	98,7 %
	**Tumor front**	7,2 %	88,1	83,3 %	22,7 %	2378,9	99,2 %
	**Tumor back**	-	-	-	22,0 %	1555,0	98,9 %
	**Tumor center**	-	-	-	21,8 %	1963,8	99,1 %
Patient 4	**Blood**	11,2 %	95,4	84,0 %	20,7 %	1460,2	98,2 %
	**Lymph node**	8,9 %	103,4	85,1 %	40,8 %	2041,0	98,7 %
	**Tumor back**	8,9 %	117,3	86,1 %	20,1 %	1952,5	98,7 %
	**Tumor front**	8,9 %	101,6	85,4 %	21,6 %	1244,1	98,5 %
	**Tumor center**	8,9 %	105,5	84,9 %	20,2 %	1546,6	98,7 %
Patient 5	**Blood**	8,6 %	87,3	82,7 %	22,8 %	622,6	97,7 %
	**Lymph node**	9,0 %	108,7	85,1 %	23,4 %	1842,3	98,9 %
	**Tumor back**	9,2 %	88,5	83,7 %	24,4 %	1472,0	98,8 %
	**Tumor front**	9,2 %	119,5	85,6 %	25,9 %	1326,4	98,8 %
	**Tumor center**	8,9 %	117,9	85,8 %	25,8 %	1697,8	98,9 %
Mean			95,1	84,1 %		1692,7	98,5 %

Whole exome sequencing and validation. Statistics for whole exome sequencing and for the validation. Duplication: proportion of duplicated sequences. Bases > 20×: proportion of bases covered by at least 20 reads. Bases > 100×: proportion of bases covered by at least 100 reads.

Supplementary Table 3: Identified variants for patient 1. See Supplementary_Table_3

Supplementary Table 4 Identified variants for patient 2. See Supplementary _Table_4

Supplementary Table 5: Identified variants for patient 3. See Supplementary _Table_5

Supplementary Table 6: Identified variants for patient 4. See Supplementary _Table_6

Supplementary Table 7: Identified variants for patient 5. See Supplementary _Table_7

Supplementary Table 8: iCAGES driver genes

Gene	Category	RadialSVM	Phenolyzer	iCAGESGeneScore
**Patient 1**				
TP53	Cancer Gene Census	1.013	1	0.999552976
PIK3CB	KEGG Cancer Pathway	1.091	0.4551	0.933716985
CBL	Cancer Gene Census	0.957	0.4165	0.819186365
BRCA1	Cancer Gene Census	0.662	0.5705	0.806267597
PTCH1	KEGG Cancer Pathway	0.986	0.326	0.681012686
LRP2	Other Category	1.196	0.1681	0.58213936
SPRY2	Other Category	0.928	0.2876	0.512107343
CALML5	Other Category	1.002	0.2279	0.464321519
CYP17A1	Other Category	1.101	0.1725	0.462072484
**Patient 2**				
TP53	Cancer Gene Census	0	1	0.8891906
MED12	Cancer Gene Census	0.972	0.1565	0.262994206
SEC31B	Other Category	1.186	0.02672	0.240264112
DMD	Other Category	0.966	0.06835	0.124153148
DCAF5	Other Category	1.054	0.01117	0.114881114
MYO16	Other Category	1.057	0.005691	0.111010461
**Patient 3**				
SOS1	KEGG Cancer Pathway	0.96	0.5	0.91454541
HLA-DRB5	Other Category	1.097	0.2392	0.622247095
PLCB1	Other Category	0.93	0.3121	0.576123349
AFP	Other Category	1.039	0.1588	0.346404933
HSPA6	Other Category	0.967	0.1689	0.282301181
FCRL2	Other Category	1.12	0.06316	0.24045582
DGKG	Other Category	0.963	0.149	0.239359397
USP8	Other Category	0.97	0.1401	0.230208282
**Patient 4**				
APC	Cancer Gene Census	0.907	0.5143	0.902052488
ERBB3	Other Category	1.017	0.3382	0.741518518
JUP	KEGG Cancer Pathway	0.984	0.2604	0.521259365
HIST4H4	Other Category	0.986	0.2423	0.478385097
PPARGC1A	Other Category	0.941	0.2276	0.381070952
DSEL	Other Category	1.159	0.0896	0.339275912
SPTBN4	Other Category	1.192	0.04753	0.287447983
MAMLD1	Other Category	0.96	0.1444	0.228058879
RPS6KA6	Other Category	0.947	0.1396	0.207508404
**Patient 5**				
TP53	Cancer Gene Census	1.005	1	0.999532661
TLN1	Other Category	1.049	0.2681	0.628098973
PPP2R5D	Other Category	0.919	0.3152	0.568820092
CXCR1	Other Category	0.98	0.2787	0.561584369
CDC20	Other Category	1.021	0.2062	0.436243974
GRM5	Other Category	0.96	0.1699	0.276501047
DGCR8	Other Category	1.135	0.06617	0.261839228
RYR2	Other Category	1.034	0.1198	0.25800392
BMP7	Other Category	0.965	0.154	0.250752138
TOP2A	Other Category	0.888	0.1941	0.246430302

Genes identified as driver genes by iCAGES based on missense mutations. RadialSVM: score measuring the driving potential of this missense mutation. Phenolyzer: score measuring its association with cancer based on prior knowledge and gene-gene interaction. iCAGESGeneScore: score measuring the final cancer driving potential.

Supplementary Table 9: Synonymous to nonsynonymous ratio

	**Patient 1**	**Patient 2**	**Patient 3**	**Patient 4**	**Patient 5**	**Overall**
Nonsynonymous (NS)	93	22	70	92	83	360
Synonymous (S)	41	3	33	31	21	129
NS + S	134	25	103	123	104	489
Ratio	2.27	7.33	2.12	2.97	3.95	2.79
P-value	0.280	0.015	0.440	0.032	0.002	0.0002
Total mutations	156	27	116	142	116	557

One would expect a ratio of 2:1 if the mutations were random passenger mutations. One-tailed binormial test was used.

Supplementary Table 10: Recurrent gene mutations. See Supplementary_Table_10

SUPPLEMENTARY NOTE 1

Construction of phylogenetic trees

The phylogenetic trees are based on the BAF vs copy number plots (Supplementary Figures 4–22) and the mutational data (Supplementarys Table 3–7). Detecting possible subclones was done by visual interpretation of the plots (Supplementary Figure 23). In a tumor, there are somatic point mutations and copy number mutations which are present in all of the cancer cells, these mutations are believed to be the earliest, and they are, in the figure, represented by grey colored circles. We expect that these early point mutations, which are still located in diploid regions (AB_{all}, A being the wildtype allele and B being the mutated allele), show a pronounced cluster at $BAF_{all} \approx ½BAF_{max}$ and $LogR \approx 0$. From this cluster the tumor content (TC) can easily be derived: $TC = 2BAF_{all}$. Correspondingly, there will be LOH regions in all cancer cells (B_{all}) in which the wildtype A-allele is lost, and the somatic point mutations in these regions are located at $BAF \approx \frac{TC}{2-TC}$ and LogR between 0 and -1 dependent on TC. Loss of wildtype and two copies of mutation allele (BB) can be identified for all cancer cells at LogR = 0 and $BAF \geq B_{all.}$ Likewise the A-allele or the B-allele can be duplicated in all tumor cells giving rise to the minor (grey) clusters marked AAB_{all} and ABB_{all} at $LogR \approx 0.58$.

With identification of the events in all cancer cells, subclonal events can be identified by locating clusters of mutations that differ from these patterns. Specific subclonal point mutations and subclonal copy number events that occur in subclonal specific mutations are represented by red colored circles. The $AB_{subclone}$ region will have a lower BAF than ½TC ($LogR \approx 0$), and its corresponding LOH region ($B_{subclone}$) will be located to the right of $AB_{subclone}$ with a LogR value between 0 and LogR of B_{all}. Loss of wildtype and two copies of mutation allele (BB) for the subclone can be located at LogR = 0 and $BAF \geq AB_{subclone}$ (not shown in figure). Duplication, of either the A-allele or B-allele, is shown to give a similar pattern for the subclone as for the early mutations in all tumor cells.

Interestingly, a subclone can alter the copy number of somatic point mutations present in all cancer cells, but the LogR will be characteristic of the subclone. Subclonal specific copy number events that occur in point mutations present in all cancer cells are represented by grey colored circles with a red outline. For a subclone that alters the copy number of somatic mutations present in all cells, $B_{subclone}$ will be located diagonally between AB_{all} and B_{all}, and $BB_{subclone}$ will have a LogR value between LogR of $B_{all} \leq 0$, and a $BAF > AB_{all}$.

Combining the approach detailed above with the use of color coding the mutations, based on which biopsy they appear in, we can determine the distribution of subclones. Mutations that characterize different subclones can be found in different biopsies with different distribution. The distribution of a clone is determined as the ratio between $BAF(AB_{subclone})$ and $BAF(AB_{all})$. Lastly, using the assumption that every clone inherits the ancestral clone's somatic mutations, we can determine the tumor evolution. Mutations believed to be the earliest are present in all biopsies, as these mutations have been inherited by all clones. These mutations would characterize the ancestral clone; however, as described above, a subclone can also be characterized by copy number events. This means that mutations that are present in all biopsies but differ from the ancestral clone would be one or more subclones. Mutations that are not present in all biopsies characterize one or more subclones. It can be difficult to determine the lineage of parallel evolved subclones as they are derived from one ancestor, therefore we cannot determine which evolved first. In contrast, linearly evolved subclones have a clear lineage.

The phylogenetic trees are constructed with the assumption that each lymph node metastasis is derived from one cell of a clone in the primary tumor. The mutations that are seen in all cancer cells in the metastasis, determines which primary tumor clone it was derived from.
